# Low c-Kit expression identifies primitive, therapy-resistant CML stem cells

**DOI:** 10.1172/jci.insight.157421

**Published:** 2023-01-10

**Authors:** Mansi Shah, Harish Kumar, Shaowei Qiu, Hui Li, Mason Harris, Jianbo He, Ajay Abraham, David K. Crossman, Andrew Paterson, Robert S. Welner, Ravi Bhatia

**Affiliations:** 1 Division of Hematology and Oncology, University of Alabama at Birmingham, Birmingham, Alabama, USA.; 2 State Key Laboratory of Experimental Hematology, National Clinical Research Center for Blood Diseases, Haihe Laboratory of Cell Ecosystem, Institute of Hematology and Blood Diseases Hospital, Chinese Academy of Medical Sciences and Peking Union Medical College, Tianjin, China.; 3 Department of Genetics and; 4 Division of Endocrinology, Diabetes and Metabolism, University of Alabama at Birmingham, Birmingham, Alabama, USA.

**Keywords:** Hematology, Oncology, Adult stem cells, Growth factors, Leukemias

## Abstract

Despite the efficacy of tyrosine kinase inhibitors (TKIs) in chronic myeloid leukemia (CML), malignant long-term hematopoietic stem cells (LT-HSCs) persist as a source of relapse. However, LT-HSCs are heterogenous and the most primitive, drug-resistant LT-HSC subpopulations are not well characterized. In normal hematopoiesis, self-renewal and long-term reconstitution capacity are enriched within LT-HSCs with low c-Kit expression (c-KIT^lo^). Here, using a transgenic CML mouse model, we found that long-term engraftment and leukemogenic capacity were restricted to c-KIT^lo^ CML LT-HSCs. CML LT-HSCs demonstrated enhanced differentiation with expansion of mature progeny following exposure to the c-KIT ligand, stem cell factor (SCF). Conversely, SCF deletion led to depletion of normal LT-HSCs but increase in c-KIT^lo^ and total CML LT-HSCs with reduced generation of mature myeloid cells. CML c-KIT^lo^ LT-HSCs showed reduced cell cycling and expressed enhanced quiescence and inflammatory gene signatures. SCF administration led to enhanced depletion of CML primitive progenitors but not LT-HSCs after TKI treatment. Human CML LT-HSCs with low or absent c-KIT expression were markedly enriched after TKI treatment. We conclude that CML LT-HSCs expressing low c-KIT levels are enriched for primitive, quiescent, drug-resistant leukemia-initiating cells and represent a critical target for eliminating disease persistence.

## Graphical abstract

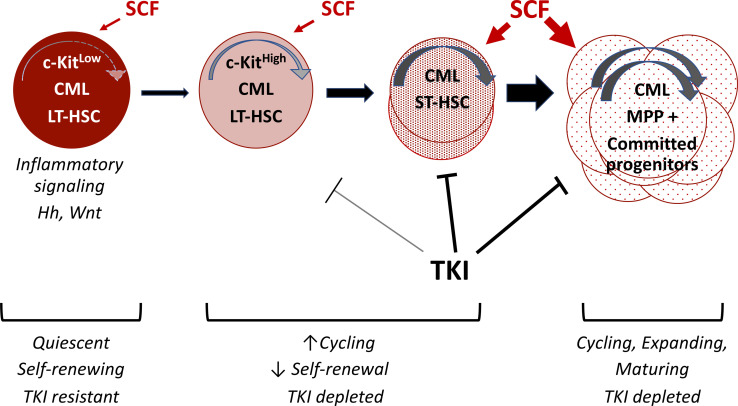

## Introduction

Chronic myelogenous leukemia (CML) results from long-term hematopoietic stem cell (LT-HSC) transformation by the *BCR-ABL1* oncogene. BCR-ABL tyrosine kinase inhibitors (TKIs) can induce deep remission and prolonged survival in patients with CML (1). However, leukemic LT-HSCs resist elimination and persist as a source of relapse in TKI-treated patients (2). Others and we have shown that CML LT-HSCs resist TKI treatment via BCR-ABL–independent mechanisms (3–5). In normal hematopoiesis, LT-HSCs demonstrate heterogeneity in differentiation and repopulation potential related to predetermined cell fate commitment (6, 7). However, CML LT-HSC heterogeneity has not been prospectively characterized, and drug-resistant LT-HSC subpopulations responsible for leukemia persistence and relapse have not been identified.

The c-Kit receptor tyrosine kinase is expressed predominately on hematopoietic stem and progenitor cells (HSPCs) and plays a crucial role in maintaining HSC function and pool size (8, 9). Stem cell factor (SCF), the ligand for c-KIT, is required for HSC maintenance in vivo (9–13). Specialized microenvironmental niches modulate LT-HSC quiescence, self-renewal, and longevity (14) and influence proliferation and lineage commitment of HSC progeny (15). SCF expression on endothelial and mesenchymal stromal niches is required for LT-HSC maintenance in adult BM (16). Interestingly, although c-Kit is required for maintaining stemness (17, 18), LT-HSCs expressing low levels of cell surface c-KIT protein (c-KIT^lo^) exhibit enhanced self-renewal and long-term reconstitution ability compared with LT-HSCs with high c-KIT expression (c-KIT^hi^) (19–21). These observations suggest that variability in c-KIT expression can differentiate heterogenous LT-HSC subpopulations.

CML HSPCs are reported to show altered responsiveness to SCF compared with their normal counterparts (22, 23). Leukemia-initiating cells in a CML blast crisis mouse model had variable c-KIT expression, whereas leukemia-initiating cells in chronic-phase CML were present in the c-KIT–positive fraction (24). Here we investigated whether levels of cell surface c-KIT expression could define subpopulations of CML LT-HSCs with heterogenous self-renewal and regenerative potential and differential response to TKI treatment. Our studies indicate that low c-KIT expression identifies a primitive, quiescent, treatment-resistant CML LT-HSC subpopulation with distinct regulatory characteristics.

## Results

### C-KIT^lo^ LT-HSCs are enriched in CML compared with normal BM.

**Figure 1:**
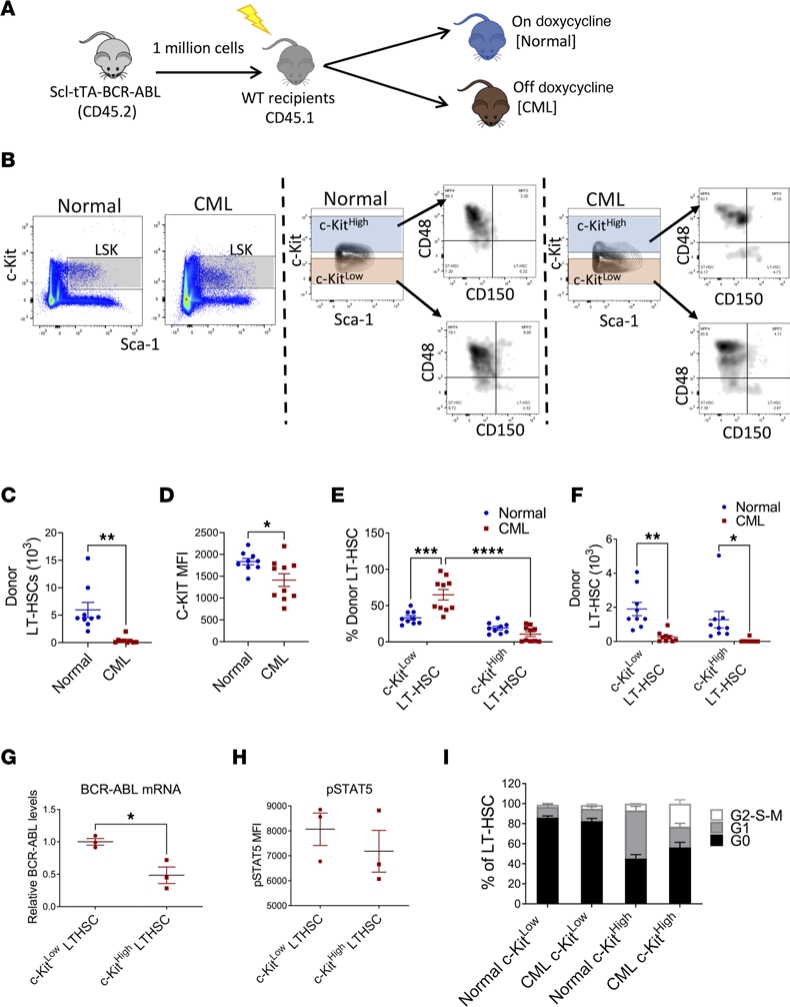
C-KIT^lo^ LT-HSCs are increased in CML compared with normal BM. Experimental design: BM cells from SCL-tTA mice (CD45.2) were transplanted into lethally irradiated recipients (CD45.1) and were kept on doxycycline (normal controls), or without doxycycline, resulting in BCR-ABL expression and development of CML (CML). Mice were analyzed 10 weeks posttransplant (**A**). Representative FACS plots showing gating strategy for c-KIT^lo^ and c-KIT^hi^ LT-HSCs in normal (left) and CML (right) mice (**B**). Total number of donor LT-HSCs in the BM (**C**). Surface c-KIT expression (MFI) on donor LT-HSC in normal and CML mice (**D**). Frequency (**E**) and absolute number (**F**) of donor c-KIT^lo^ and c-KIT^hi^ LT-HSCs in normal and CML mice (*n* = 9–10). qRT-PCR analysis of *Bcr-Abl1* mRNA expression in donor c-KIT^lo^ and c-KIT^hi^ LT-HSCs in CML mice (**G**). Results are expressed relative to GAPDH and normalized to c-KIT^lo^ LT-HSCs. Levels of p-STAT5 expression in c-KIT^lo^ and c-KIT^hi^ LT-HSCs from CML mice measured by flow cytometry after intracellular labeling with anti–p-STAT5 antibody (**H**). Cell cycle analysis of freshly isolated BM cells showing percentage of G_0_, G_1_, and S-G_2_-M phase normal and CML c-KIT^lo^ and c-KIT^hi^ LT-HSCs (*n* = 4 each) (**I**). Compiled data are presented as mean ± SEM, *P < 0.05, **P < 0.01, ***P < 0.001, ****P < 0.0001, based on *t* test (**C**, **D**, and **H**) and 2-way ANOVA with Tukey’s test (**E** and **F**).

Previous studies indicate that c-KIT^lo^ LT-HSCs represent a primitive, quiescent subset with increased multi-lineage-repopulating ability (19–21). We studied c-KIT expression in CML LT-HSCs using a well-characterized transgenic Tg(Tal1-tTA)19Dgt × Tg(tetO-BCR/ABL1)2Dgt (Scl-tTA BCR-ABL) mouse model of CML. In these mice, withdrawal of tetracycline induces *BCR-ABL1* expression and development of a CML-like myeloproliferative disorder. BM cells from CML mice (CD45.2) were transplanted into lethally irradiated wild-type recipients (CD45.1) to generate cohorts with similar time of onset of CML (Supplemental Figure 1, A–C; supplemental material available online with this article; https://doi.org/10.1172/jci.insight.157421DS1). A control group was maintained on doxycycline (Figure 1A). LT-HSCs (Lin^–^Sca-1^+^c-KIT^+^Flt3^–^CD150^+^CD48^–^) present within donor Lin^–^Sca-1^+^c-KIT^+^ (LSK) cells in control mice with the highest c-KIT expression (top 30%) were defined as c-KIT^hi^ LT-HSCs, whereas LT-HSCs with the lowest c-KIT expression (bottom 30%) were defined as c-KIT^lo^ LT-HSCs (Figure 1B). As previously described, total LT-HSC frequency and numbers were significantly reduced in BM from CML mice (Figure 1C and Supplemental Figure 1D) (25, 26). The mean fluorescence intensity (MFI) of c-KIT was significantly decreased in CML compared with control LT-HSCs (Figure 1D). Application of gates established for normal c-KIT^hi^ and c-KIT^lo^ cells to CML LT-HSCs (Figure 1B) showed increased proportions of c-KIT^lo^ LT-HSCs and reduced proportions of c-KIT^hi^ LT-HSCs in CML mice (Figure 1E), despite reduction in absolute numbers of c-KIT^lo^ LT-HSCs (Figure 1F). Therefore, CML development, though leading to depletion of LT-HSCs, is associated with proportionately greater retention of c-KIT^lo^ LT-HSCs.

Quantitative reverse transcriptase PCR (qRT-PCR) analysis showed no significant difference in *c-Kit* mRNA expression between c-KIT^lo^ and c-KIT^hi^ LT-HSCs from normal and CML mice (Supplemental Figure 1E). To determine c-KIT localization, cell surface c-KIT was stained at saturation levels, and cells were fixed and permeabilized to separately label intracellular c-KIT. As with cell surface c-KIT, intracellular c-KIT was significantly lower in CML compared with normal LT-HSCs (Supplemental Figure 1F). Intracellular c-KIT expression was reduced in c-KIT^lo^ CML compared with normal LT-HSCs but similar in normal and CML c-KIT^hi^ LT-HSCs (Supplemental Figure 1G). These results suggest that reduced surface c-KIT levels in CML LT-HSCs are not related to reduced RNA expression or protein relocalization to the intracellular compartment but could reflect increased c-KIT protein degradation, as was reported for normal c-KIT^lo^ LT-HSCs (21). We also evaluated *Bcr-Abl1* mRNA expression in CML KIT^lo^ and c-KIT^hi^ LT-HSCs using qRT-PCR (Figure 1G) and BCR-ABL signaling by measuring phosphorylated STAT5 (p-STAT5) levels in CML KIT^lo^ and c-KIT^hi^ LT-HSCs using intracellular flow cytometry (Figure 1H). CML KIT^lo^ LT-HSCs expressed higher levels of *Bcr-Abl1* mRNA, but did not express significantly higher levels of p-STAT5 levels, than c-KIT^hi^ LT-HSCs.

We assessed cell cycling using DAPI and Ki-67 labeling (Figure 1I). Normal c-KIT^lo^ LT-HSCs were highly quiescent and predominantly in G_0_. Normal c-KIT^hi^ LT-HSCs showed substantially reduced proportions of cells in G_0_ and increased proportions of cells in G_1_ compared with normal c-KIT^lo^ LT-HSCs, suggesting a more activated state. CML c-KIT^lo^ LT-HSCs showed similarly high proportions of cells in G_0_ as normal KIT^lo^ LT-HSCs, indicating that they were also highly quiescent. CML c-KIT^hi^ LT-HSCs showed substantially reduced proportions of cells in G_0_ and increased proportions of cells in S-G_2_-M compared with CML c-KIT^lo^ LT-HSCs. CML c-KIT^hi^ LT-HSCs showed reduced G_1_ phase and increased G_2_-S-M phase, compared with normal c-KIT^hi^ LT-HSCs, suggesting increased progression through the cell cycle.

### CML c-KIT^lo^ LT-HSCs exhibit gene signatures characteristic of primitive drug-resistant leukemia stem cells.

**Figure 2:**
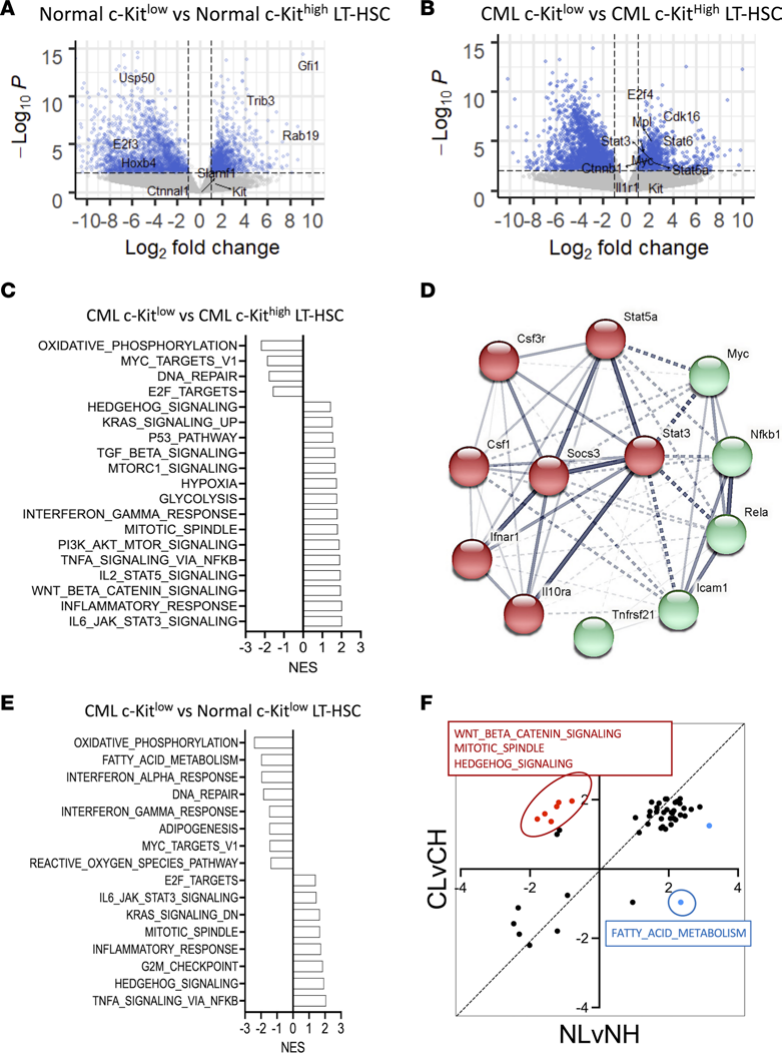
CML c-KIT^lo^ LT-HSCs exhibit gene signatures characteristic of primitive, drug-resistant leukemia stem cells. Volcano plots of differential expression of normal c-KIT^lo^ LT-HSCs versus normal c-KIT^hi^ LT-HSCs (**A**) and CML c-KIT^lo^ LT-HSCs versus CML c-KIT^hi^ LT-HSCs (*n* = 3 each) (**B**). Known HSC drivers and markers are indicated. Normalized enrichment scores (NES) of Hallmark gene sets significantly enriched (FDR < 0.05) in CML c-KIT^lo^ LT-HSCs compared with CML c-KIT^hi^ LT-HSCs (**C**). Protein-protein association network analysis of leading-edge genes from the inflammation related gene sets enriched in CML c-KIT^lo^ LT-HSCs compared with CML c-KIT^hi^ LT-HSCs was performed using STRING (**D**). NES of Hallmark gene sets significantly enriched (FDR < 0.05) in CML c-KIT^lo^ LT-HSCs compared with normal c-KIT^lo^ LT-HSCs (**E**). Dot plot of NES of Hallmark gene sets significantly enriched (FDR < 0.05) in CML c-KIT^lo^ LT-HSCs compared with CML c-KIT^hi^ LT-HSCs (CL vs. CH) and normal c-KIT^lo^ LT-HSCs compared to normal c-KIT^hi^ LT-HSCs (NL vs. NH). Gene sets that are selectively enriched in CML c-KIT^lo^ LT-HSCs but not normal c-KIT^lo^ LT-HSCs are indicated in red (positively enriched) and blue (negatively enriched) (**F**).

RNA-Seq analysis was performed on normal c-KIT^hi^, normal c-KIT^lo^, CML c-KIT^hi^, and CML c-KIT^lo^ LT-HSCs. We compared gene expression between (a) normal c-KIT^hi^ and c-KIT^lo^ LT-HSCs, (b) CML c-KIT^hi^ and c-KIT^lo^ LT-HSCs, and (c) normal and CML c-KIT^lo^ LT-HSCs. Increased expression of several HSC-associated genes, including *Trib3* and *Gfi1*, was seen in normal c-KIT^lo^ versus c-KIT^hi^ LT-HSCs (Figure 2A). Increased expression of several genes previously reported to be associated with CML stem cells and TKI resistance, including *Myc*, *Mpl*, *Stat3*, *Stat5a*, *Stat6*, and *Ctnnb*, was seen in CML c-KIT^lo^ versus c-KIT^hi^ LT-HSCs (Figure 2B) (27–34). Interestingly, we have previously shown that CML LT-HSCs with higher levels of MPL expression have increased leukemogenic capacity and reduced TKI sensitivity (35).

Gene set enrichment analysis (GSEA) comparing normal c-KIT^lo^ and normal c-KIT^hi^ LT-HSCs showed that normal c-KIT^lo^ LT-HSCs were enriched for Hallmark gene signatures for interferon signaling, hypoxia, glycolysis, lipid metabolism, p53 signaling, and inflammatory signaling (STAT3, STAT5, TNFA), whereas normal c-KIT^hi^ LT-HSCs were enriched for oxidative phosphorylation (OXPHOS), cell cycle, MYC, and Hedgehog gene signatures (Supplemental Figure 2A). GSEA comparing CML c-KIT^lo^ and CML c-KIT^hi^ LT-HSCs showed that the latter were enriched for gene signatures for inflammatory signaling (IL-6/JAK/STAT3, IL-2/STAT5, and TNF-α/NF-κB signaling; inflammatory response); TGF-β, Wnt/β-catenin, and Hedgehog signaling; hypoxia; and glycolysis, whereas CML c-KIT^hi^ LT-HSCs were enriched for OXPHOS, Myc and E2F targets, and DNA repair (Figure 2C). Enrichment of MYC gene signatures in CML c-KIT^hi^ LT-HSCs despite reduced Myc mRNA expression could reflect posttranscriptional regulation or modulation of MYC transcriptional activity. Analysis of C2 signatures showed enrichment of inflammatory and Wnt/β-catenin signaling signatures in CML c-KIT^lo^ LT-HSCs and OXPHOS and cell proliferation signatures in CML c-KIT^hi^ LT-HSCs (Supplemental Table 1). Further analysis of genes contributing to inflammatory signaling profiles in CML c-KIT^lo^ LT-HSCs revealed increased expression of *Csf1*, *Tnfrsf21* (DR6), IL-10 receptor, CSF3 (G-CSF) receptor, and IFN-α receptor, together with increased expression of *Stat3*, *Stat5*, *Socs3*, *NF-kB*, and *c-Myc* (Figure 2D and Supplemental Figure 2B).

GSEA comparing CML c-KIT^lo^ LT-HSCs and normal c-KIT^lo^ LT-HSCs showed that CML c-KIT^lo^ LT-HSCs were enriched for inflammatory signaling (TNF-α/NF-κB, IL-6/JAK/STAT3; inflammatory response), Hedgehog signaling, and cell cycle (G2M checkpoint, mitotic spindle, E2F targets) signatures, whereas normal c-KIT^lo^ LT-HSCs were enriched for OXPHOS, fatty acid metabolism, and interferon signatures (Figure 2E). Analysis of the C2 signatures data set validated enrichment of inflammatory signaling and cell cycling signatures in CML c-KIT^lo^ LT-HSCs and OXPHOS and interferon pathway signatures in normal c-KIT^lo^ LT-HSCs (Supplemental Table 1). Leading edge analysis of inflammatory signaling profiles revealed increased *Socs3*, *Stat3*, *Stat5*, and *NF-kB* expression in CML compared with normal c-KIT^lo^ LT-HSCs (Supplemental Figure 2B).

Finally, we compared gene signatures enriched in c-KIT^lo^ versus c-KIT^hi^ normal LT-HSCs (FDR < 0.05) with signatures enriched in c-KIT^lo^ versus c-KIT^hi^ CML LT-HSCs (FDR < 0.05) (Figure 2F). Inflammatory signaling, glycolysis, and hypoxia signatures were enriched in both normal and CML c-KIT^lo^ LT-HSCs compared with their c-KIT^hi^ LT-HSC counterparts, whereas OXPHOS, Myc, and cell cycle signatures were enriched in both normal and CML c-KIT^hi^ LT-HSCs compared with their c-KIT^lo^ LT-HSC counterparts (Figure 2F). On the other hand, signatures for Wnt and Hedgehog signaling, important mechanisms regulating primitive leukemia stem cells (LSCs) in CML (5, 34, 36, 37), were selectively enriched in CML c-KIT^lo^ versus c-KIT^hi^ LT-HSCs, and fatty acid metabolism signatures were selectively enriched in normal c-KIT^lo^ versus c-KIT^hi^ LT-HSCs.

In conclusion, CML c-KIT^lo^ LT-HSCs showed reduced cell cycling signatures and increased STAT3 and NF-κB gene signatures, which are gene expression characteristics similar to those reported for BCR-ABL^+^CD34^+^ cell subpopulations enriched in patients with CML following TKI treatment (38).

### CML c-KIT^lo^ LT-HSCs are enriched for long-term engraftment and leukemia-generating capacity.

**Figure 3:**
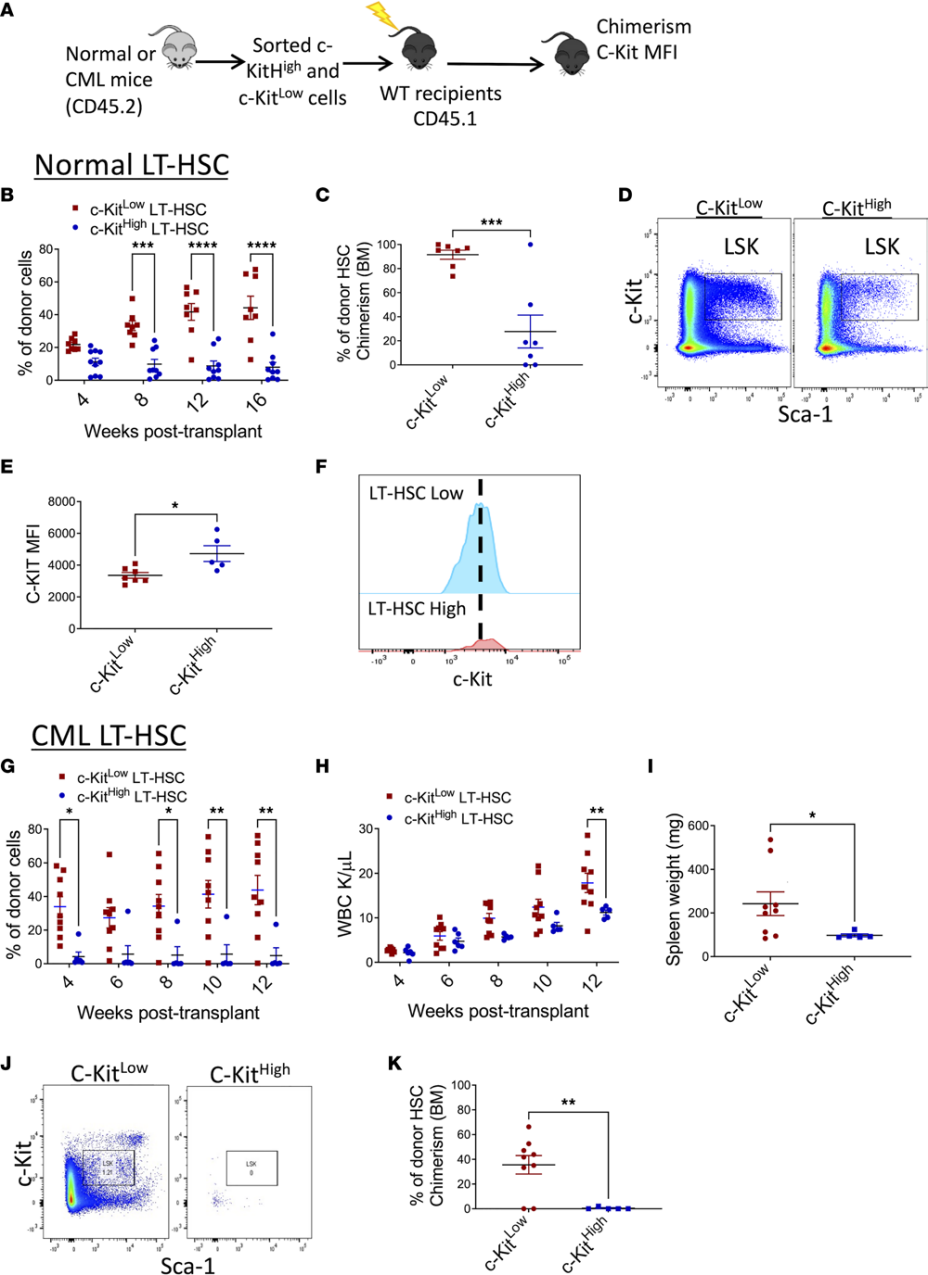
Long-term repopulating and disease-generating capacity is restricted to CML c-KIT^lo^ LT-HSCs. FACS-sorted c-KIT^hi^ and c-KIT^lo^ LT-HSCs from both normal and CML mice (CD45.2) (150 LT-HSCs together with 200,000 support BM cells [CD45.1]) were transplanted into lethally irradiated recipient (CD45.1) mice and followed for donor chimerism (**A**). Peripheral blood (PB) normal donor chimerism following transplantation of normal c-KIT^lo^ LT-HSCs (*n* = 8) and c-KIT^hi^ LT-HSCs (*n* = 9) (**B**). Representative FACS plots showing donor LSK cells in transplant recipients of normal c-KIT^lo^ LT-HSCs and c-KIT^hi^ LT-HSCs (**C**). Percentage of donor LT-HSC chimerism at 16 weeks following transplantation of normal c-KIT^lo^ LT-HSCs and c-KIT^hi^ LT-HSCs (**D**). MFI of surface c-KIT levels in donor LSK cells in recipient mice transplanted with normal c-KIT^lo^ LT-HSCs and c-KIT^hi^ LT-HSCs (**E**). Concatenated histogram plots showing c-KIT MFI on donor LT-HSCs in recipient mice transplanted with c-KIT^lo^ LT-HSCs and c-KIT^hi^ LT-HSCs (**F**). PB normal donor chimerism (**G**) and white blood count (WBC) (**H**) following transplantation of CML c-KIT^lo^ LT-HSCs (*n* = 8) and c-KIT^hi^ LT-HSCs (*n* = 5). Spleen weights in recipient mice 16 weeks following transplantation of CML c-KIT^lo^ LT-HSCs and c-KIT^hi^ LT-HSCs (**I**). Representative FACS plots showing donor LSK cells in recipients of CML c-KIT^lo^ LT-HSCs and c-KIT^hi^ LT-HSCs (**J**). Percentage of donor LT-HSC chimerism at 16 weeks following transplantation of CML c-KIT^lo^ LT-HSCs and c-KIT^hi^ LT-HSCs (**K**). Data represented as mean ± SEM, *P < 0.05, **P < 0.01, ***P < 0.001, ****P < 0.0001, based on 2-way ANOVA with Holm-Šídák test (**B**, **G**, and **H**) and *t* test (**C**, **E**, **I**, and **K**).

We transplanted FACS-purified c-KIT^hi^ and c-KIT^lo^ LT-HSCs (CD45.2) from CML and normal mice into lethally irradiated recipients (CD45.1) to evaluate their repopulating capacity (Figure 3A). Normal c-KIT^lo^ LT-HSCs generated significantly higher peripheral blood (PB) chimerism (Figure 3B), and BM LT-HSC chimerism, compared with c-KIT^hi^ LT-HSC (Figure 3C). LT-HSCs regenerated from c-KIT^lo^ LT-HSCs showed significantly lower c-KIT MFI than LT-HSCs regenerated from c-KIT^hi^ LT-HSCs, indicating regeneration of c-KIT^lo^ LT-HSCs after transplantation (Figure 3, D–F). Following transplantation, long-term donor chimerism was restricted to CML c-KIT^lo^ LT-HSCs, whereas CML c-KIT^hi^ LT-HSCs generated minimal engraftment (Figure 3G). Leukemia induction in recipient mice, as indicated by increased WBC counts (Figure 3H) and increased spleen weight (Figure 3I), was restricted to CML c-KIT^lo^ LT-HSCs. CML c-KIT^lo^ LT-HSCs regenerated donor LSK cells in recipient mice, whereas CML c-KIT^hi^ LT-HSCs failed to regenerate donor LT-HSCs (Figure 3, J and K), and their c-KIT MFI could not be determined. These results indicate that primitive LSCs capable of long-term engraftment and leukemia generation are restricted to CML c-KIT^lo^ LT-HSCs, whereas c-KIT^hi^ LT-HSCs lack long-term repopulating and leukemia-generating capacity.

### SCF stimulation drives enhanced differentiation and expansion of mature progeny from CML compared with normal LT-HSCs.

**Figure 4:**
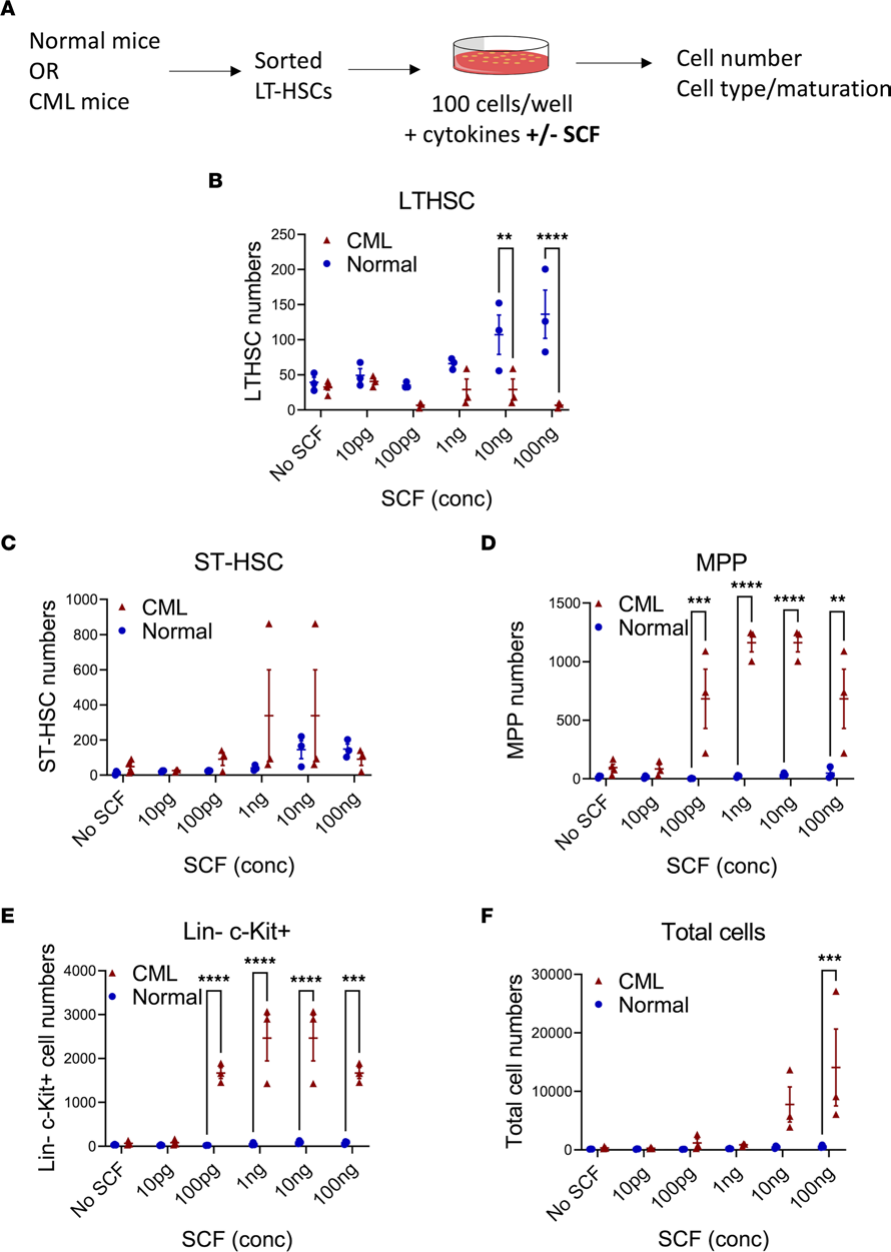
Differential response of normal and CML LT-HSCs to SCF. Experimental design: LT-HSCs isolated from normal and CML mice by flow cytometry were cultured with varying concentrations of SCF (10 pg to 100 ng) and cell numbers and cell phenotypes analyzed by flow cytometry (**A**). Number of cells with LT-HSC (**B**), ST-HSC (**C**), MPP (**D**), and LSK (**E**) phenotype, and total number of cells (**F**), generated from normal and CML LT-HSCs cultured for 6 days with increasing SCF concentrations. Normal *n* = 3 from 3–5 mice; CML *n* = 3 from 3–5 mice. Data represented as mean ± SEM, **P < 0.01, ***P < 0.001, ****P < 0.0001, based on 2-way ANOVA with Holm-Šídák test.

We analyzed the response of normal and CML LT-HSCs to SCF (0–100 ng/mL) stimulation in vitro (Figure 4A and Supplemental Figure 3A). For normal LT-HSCs, SCF exposure led to a dose-dependent increase in LT-HSCs and short-term HSCs (ST-HSCs) but lesser expansion of multipotent progenitors (MPPs), committed progenitors (Lin^–^Sca-1^–^c-KIT^+^), and total cell numbers (Figure 4, B–F). In contrast, exposure of CML LT-HSCs to SCF did not lead to significant expansion of LT-HSCs and ST-HSCs but led to significantly greater expansion of MPPs, committed progenitors, and total cell numbers compared with normal LT-HSCs (Figure 4, B–F). Therefore, SCF stimulation expands normal but not CML LT-HSCs and ST-HSCs and drives enhanced CML LT-HSC maturation and increased generation of progenitors and mature cells.

**Figure 5:**
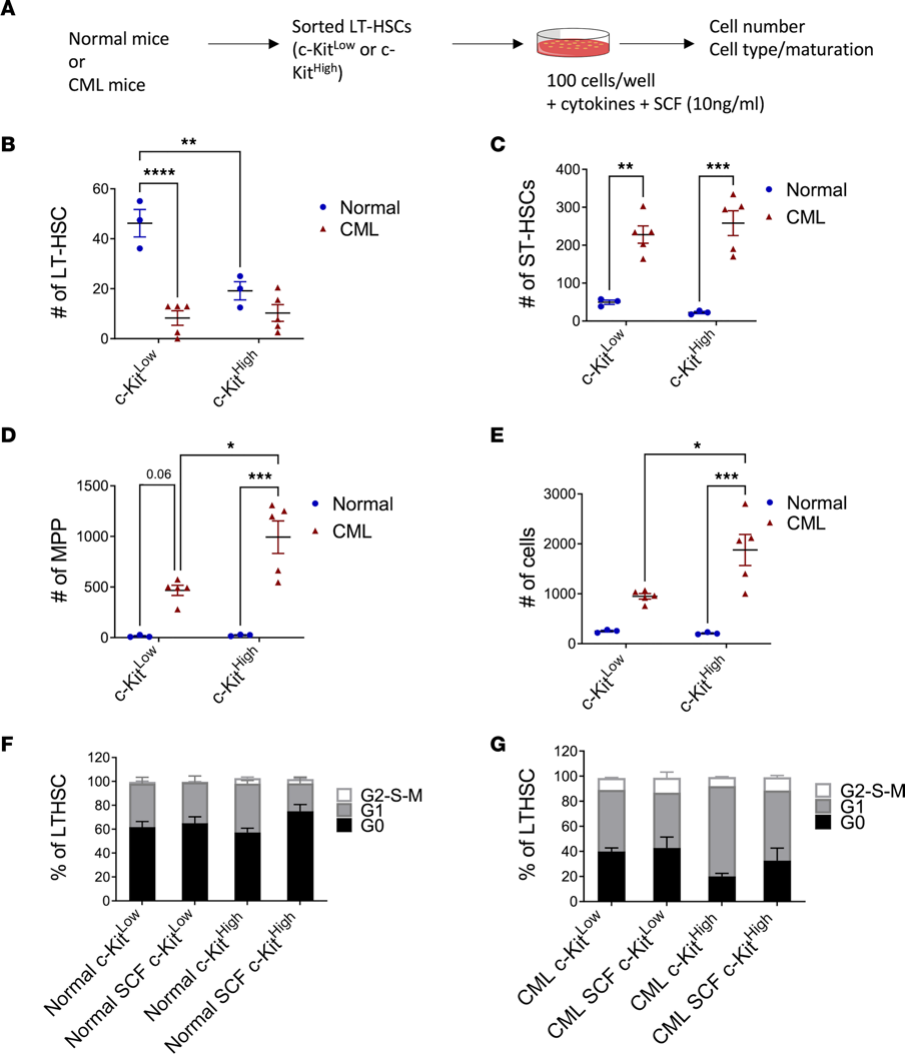
Differential response of c-KIT^lo^ normal and CML LT-HSCs to SCF. Experimental plan: c-KIT^lo^ and c-KIT^hi^ LT-HSCs isolated from normal and CML mice by flow cytometry were cultured with SCF (10 ng) and cell numbers and cell phenotypes analyzed by flow cytometry (**A**). Number of cells with LT-HSC (**B**), ST-HSC (**C**), or MPP (**D**) phenotype, and total number of cells (**E**), generated from normal and CML c-KIT^lo^ and c-KIT^hi^ LT-HSCs cultured with 10 ng SCF for 10 days (normal, *n* = 3, and CML, *n* = 5). Cell cycle analysis showing percentage of G_0_, G_1_, and S-G_2_-M phase normal (**F**) and CML (**G**) c-KIT^lo^ and c-KIT^hi^ LT-HSCs after culture for 24 hours in medium with and without SCF (*n* = 4 each). Data represented as mean ± SEM, *P < 0.05; **P < 0.01; ***P < 0.001; ****P < 0.0001, based on 2-way ANOVA with Tukey’s test.

We next compared the response of c-KIT^lo^ and c-KIT^hi^ LT-HSCs to SCF (10 ng/mL) (Figure 5A). Increased numbers of LT-HSCs (Figure 5B) and ST-HSCs (Figure 5C), and similar numbers of MPPs and total cells (Figure 5, D and E), were seen after culture of normal c-KIT^lo^ LT-HSCs with SCF compared with normal c-KIT^hi^ LT-HSCs. CML c-KIT^hi^ LT-HSCs generated increased numbers of MPPs and total cells compared with CML c-KIT^lo^ LT-HSCs (Figure 5, D and E). Significantly reduced numbers of LT-HSCs but significantly higher numbers of ST-HSCs, MPPs, and total cells was seen after culture of CML c-KIT^lo^ LT-HSCs with SCF compared with normal c-KIT^lo^ LT-HSCs (Figure 5, B–E). CML c-KIT^hi^ LT-HSCs also generated higher numbers of ST-HSCs, MPPs, and total cells after culture with SCF compared with normal c-KIT^hi^ LT-HSCs (Figure 5, C–E). We conclude that SCF stimulation leads to reduced retention of LT-HSCs but increased generation of ST-HSCs and MPPs from CML compared with normal c-KIT^lo^ LT-HSCs.

We studied the effect of SCF stimulation on cell cycling of normal (Figure 5F) and CML (Figure 5G) c-KIT^lo^ and c-KIT^hi^ LT-HSCs. Interestingly, treatment with SCF for 24 hours did not enhance cycling of c-KIT^lo^ or c-KIT^hi^ LT-HSCs. These observations suggest that SCF mediated reduction in LT-HSCs and that expansion of ST-HSCs, MPPs, and total cell numbers may not be mediated by enhanced LT-HSC cycling.

### SCF deletion enhances retention of CML c-KIT^lo^ LT-HSCs but reduces normal LT-HSCs.

SCF expressed in the BM microenvironment regulates HSC numbers and functionality (16). We sought to determine the role of in vivo SCF expression in regulating CML c-KIT^lo^ and c-KIT^hi^ LT-HSC populations. Normal and CML BM cells showed similar SCF protein levels in normal and CML BM plasma measured by ELISA and similar SCF mRNA expression in BM cells measured by qRT-PCR (Supplemental Figure 4, A and B). Using mice with an EGFP reporter targeted to the SCF locus, we found that EGFP was expressed primarily by nonhematopoietic CD45-negative cells in CML BM. The proportion of SCF-expressing CD45-negative cells was higher in CML compared with normal BM. The proportion of SCF-expressing CD45-positive cells was lower in CML compared with normal BM (data not shown). We deleted SCF expression by crossing Scf^fl/fl^ mice with UBC-Cre lines to generate Scf^fl/fl^ Ubc-cre (Cre^+^) mice, then treating mice with tamoxifen to induce *Scf* deletion (16). *Scf* excision was confirmed by genomic PCR and lack of Scf mRNA expression by qRT-PCR (Supplemental Figure 4, C and D). *Scf*-deleted mice showed significantly decreased BM cellularity, and reduced BM LT-HSCs, ST-HSCs, MPPs, granulocyte-macrophage progenitors (GMPs), and megakaryocyte-erythroid progenitors (MEPs), compared with Cre^–^ controls (Supplemental Figure 4, E–K).

**Figure 6:**
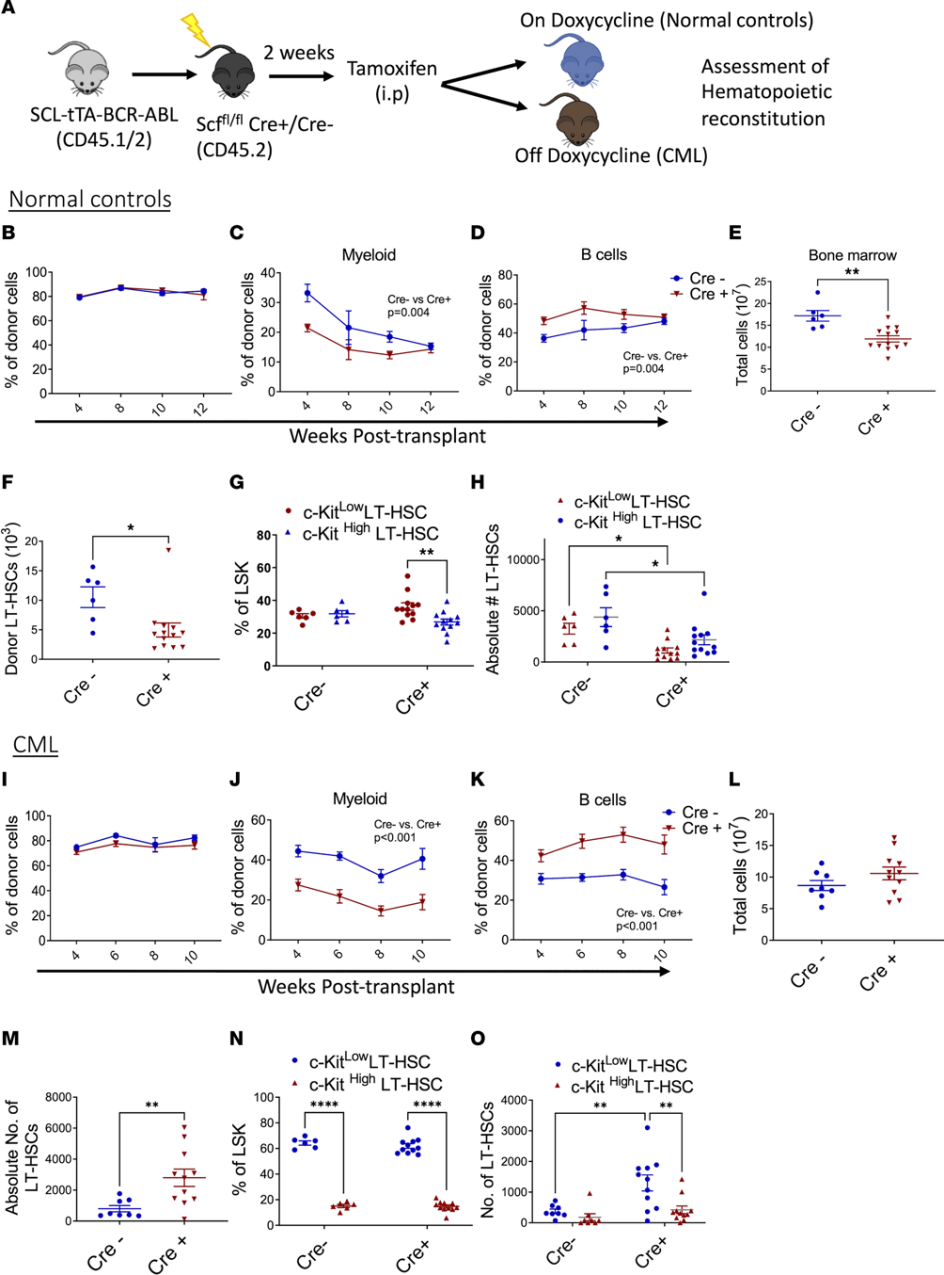
Deletion of SCF alters c-KIT^lo^ LT-HSC numbers in vivo. Experimental design: 1 × 10^6^ cells from SCL-tTA BCR-ABL mice were transplanted into lethally irradiated Scf^fl/fl^-UBC-cre mice. Cre excision was induced with tamoxifen injections. Mice were subsequently kept on tetracycline (normal controls), or off doxycycline, to allow BCR-ABL expression and CML development (CML). (**A**). Total donor chimerism (**B**) and myeloid (**C**) and B cell (**D**) chimerism in PB of normal controls. Total BM cellularity (**E**) and number of BM LT-HSCs (**F**) in normal mice. Frequency of c-KIT^lo^ and c-KIT^hi^ LT-HSCs within the LSK compartment (**G**) and number of c-KIT^lo^ and c-KIT^hi^ LT-HSCs (**H**) in BM of normal mice (Cre^–^ *n* = 6; Cre^+^ *n* = 13). Total donor chimerism (**I**) and myeloid (**J**) and B cell (**K**) chimerism in PB of CML mice. Total CML BM cellularity (**L**) and number of CML BM LT-HSCs (**M**) in CML mice. Frequency of c-KIT^lo^ and c-KIT^hi^ LT-HSCs within the LSK compartment (**N**), and number of c-KIT^lo^ and c-KIT^hi^ LT-HSC (**O**) in BM of CML mice (Cre^–^ *n* = 8; Cre^+^ *n* = 11). Data represented as mean ± SEM, *P < 0.05, **P < 0.01, ****P < 0.0001, based on 2-way ANOVA with Tukey’s test (**C**, **D**, **G**, **H**, **J**, **K**, **N**, and **O**) and *t* test (**E**, **F**, and **M**).

We next transplanted BM cells from Scl-tTA-BCR-ABL mice into lethally irradiated Cre^+^ and Cre^–^ mice and induced *Scf* deletion 2 weeks posttransplant (Figure 6A). Control mice were maintained on doxycycline, to suppress BCR-ABL expression, throughout the experiment. Our previous studies have shown that the phenotype and function of BM mesenchymal cells from irradiated mice are similar to nonirradiated mice by 4 weeks after exposure (39). Total donor chimerism was similar in PB of Cre^–^ and Cre^+^ (*Scf-*deleted) control mice, but myeloid donor chimerism was reduced and B cell donor chimerism increased in PB of Cre^+^ mice (Figure 6, B–D). BM cellularity and donor LT-HSC numbers were significantly reduced in *Scf*-deleted mice (Figure 6, E and F). The proportion of c-KIT^hi^ compared with c-KIT^lo^ donor LT-HSCs was increased in *Scf*-deleted mice (Figure 6G). Both c-KIT^lo^ and c-KIT^hi^ LT-HSC numbers were reduced in *Scf*-deleted mice (Figure 6H). To assess effects of *Scf* deletion on CML stem cells, doxycycline was withdrawn 4 weeks posttransplant to induce BCR-ABL expression (Figure 6A). Although total chimerism was not changed, significantly decreased myeloid and increased lymphoid chimerism were seen in *Scf*-deleted mice (Figure 6, I–K). Total BM cellularity and MPP and GMP numbers were not significantly altered (Figure 6L and Supplemental Figure 4, L and M). However, LT-HSC numbers were significantly increased in *Scf*-deleted CML mice compared with control Cre^–^ CML mice (Figure 6M), with a significant increase in numbers of c-KIT^lo^ compared with c-KIT^hi^ LT-HSCs (Figure 6, N and O). A significant reduction in splenic LT-HSC, MPP, and GMP numbers was observed in *Scf-*deleted mice (Supplemental Figure 4, N–P). Therefore, deletion of *Scf* reduces c-KIT^lo^ LT-HSCs, total LT-HSC and progenitor numbers, and BM cellularity in normal mice, but increases c-KIT^lo^ LT-HSCs and total LT-HSCs in CML mice, while reducing splenic progenitors and circulating myeloid cells. Increased retention of CML LT-HSCs after deletion of *Scf* from the BM environment and reduced generation of splenic progenitors and mature myeloid cells are consistent with our observations of reduced LT-HSCs and enhanced generation of mature progeny after SCF stimulation. Similarly, depletion of normal LT-HSCs after *Scf* deletion is consistent with their enhanced maintenance after SCF stimulation.

### Effect of TKI treatment on CML c-KIT^lo^ LT-HSCs.

**Figure 7:**
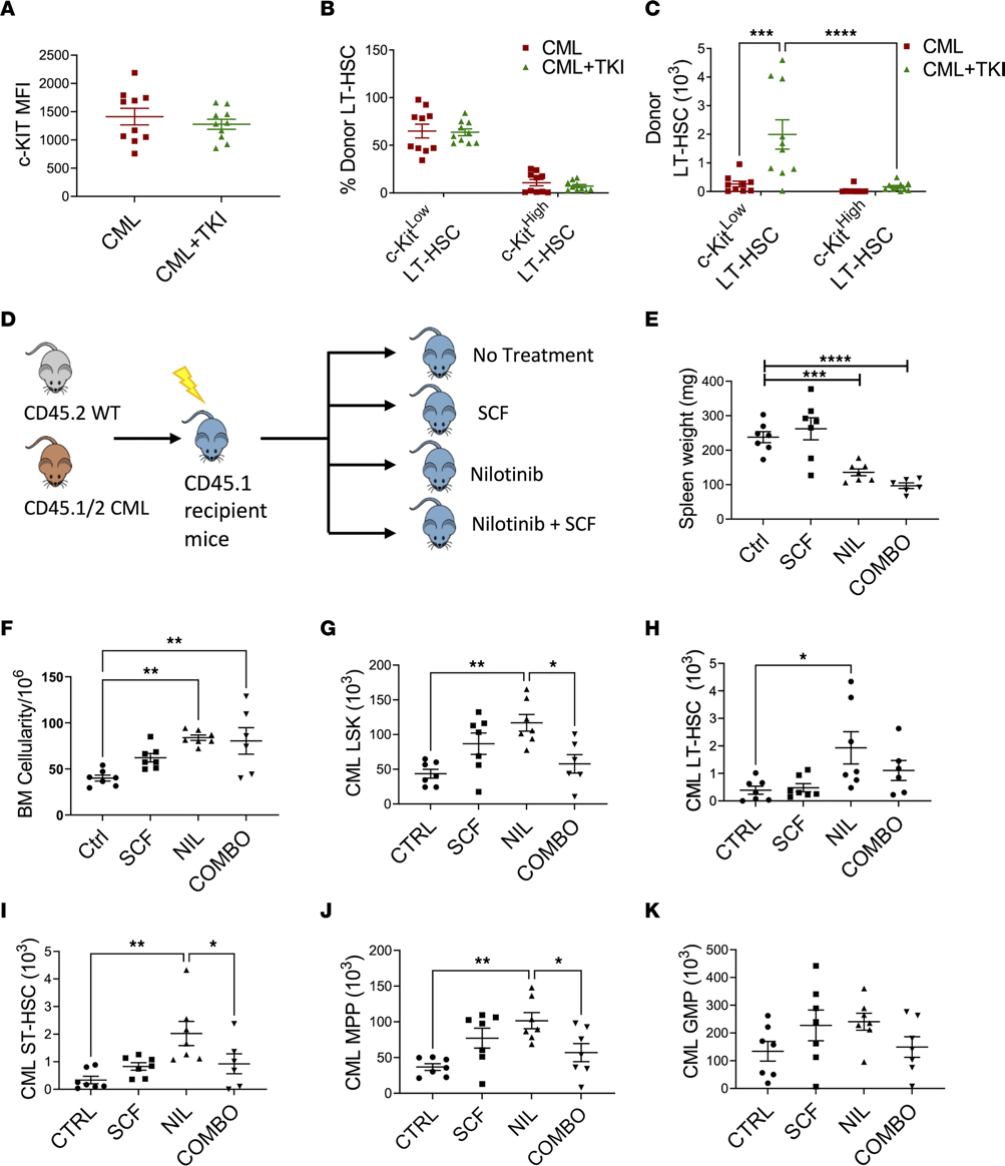
Effect of TKI treatment on murine leukemic LT-HSCs. BM cells from SCL-tTA mice (CD45.2) were transplanted into lethally irradiated recipients (CD45.1) and maintained without doxycycline, resulting in development of CML after 8 weeks, then treated with vehicle (CML) or nilotinib (50 mg/kg/d) (CML+TKI) for 14 days. MFI of surface c-KIT levels on donor LT-HSC cells (**A**) and frequency (**B**) and absolute number (**C**) of donor c-KIT^lo^ and c-KIT^hi^ LT-HSCs in CML and CML+TKI mice (*n* = 9–10). Experimental design: 1 × 10^6^ CML (CD45.1/CD45.2) and normal (CD45.2) BM cells were transplanted into lethally irradiated recipient mice (CD45.1). After 8 weeks, mice were treated with vehicle (Ctrl), SCF (100 μg/kg/d), nilotinib (50 mg/kg/d) (NIL) or SCF and nilotinib combination (COMBO) for 14 days (**D**). Spleen weights (**E**), total BM cellularity (**F**), BM CML LSK cells (**G**), BM CML LT-HSCs (**H**), BM CML ST-HSCs (**I**), BM CML MPP (**J**), and BM CML GMP (**K**) numbers after treatment (*n* = 6–7 per arm). Data represented as mean ± SEM, *P < 0.05, **P < 0.01, ***P < 0.001, ****P < 0.0001, based on 2-way ANOVA with Tukey’s test (**C**) and 1-way ANOVA (**E**–**J**).

We assessed the effects of treatment with the BCR-ABL TKI nilotinib on CML c-KIT^lo^ LT-HSCs. CML mice treated with nilotinib for 2 weeks showed reduced PB WBC and spleen cellularity, but increased BM cellularity (Supplemental Figure 5, A and B) and BM LT-HSC frequency and numbers (Supplemental Figure 5, C and D), compared with vehicle-treated mice. The c-KIT MFI in LT-HSCs was not significantly different between TKI-treated and vehicle-treated mice (Figure 7A). Although the proportion of c-KIT^lo^ LT-HSCs was not changed after TKI treatment (Figure 7B), a significant increase in c-KIT^lo^ and c-KIT^hi^ LT-HSC numbers was observed (Figure 7C).

Since our results suggested that culture with SCF was associated with increased generation of mature progeny from CML LT-HSCs, we evaluated the effects of SCF treatment in vivo on CML LT-HSC response to TKI treatment. BM cells from CML and normal mice were transplanted into lethally irradiated mice, and after 8 weeks mice were treated with nilotinib, SCF, or a combination of nilotinib and SCF for 2 weeks (Figure 7D). Both nilotinib- and combination-treated mice showed decreased circulating neutrophils compared with controls (Supplemental Figure 5E). Nilotinib markedly reduced spleen weight (Figure 7E and Supplemental Figure 5F) but significantly increased BM cellularity and donor LSK cells, LT-HSCs, ST-HSCs, and MPPs (*P* < 0.05) compared with controls (Figure 7, F–K). Compared with mice treated with nilotinib alone, mice treated with SCF in combination with nilotinib showed significantly reduced LSK cell numbers, with reduced ST-HSC and MPP numbers (Figure 7, I and J). However, reduction in LT-HSC numbers was not statistically significant (Figure 7H). Nilotinib- and combination-treated mice showed a significant reduction in splenic GMP, without significant change in splenic LSK cells, LT-HSCs, ST-HSCs, and MPPs (Supplemental Figure 5, G–K). Combination treatment did not alter normal LSK cell and progenitor populations when compared to nilotinib, but combination treatment compared with nilotinib reduced ST-HSC counts (Supplemental Figure 5, L–O). These results indicate that nilotinib treatment reduces disease burden but enhances retention of CML stem cells. Short-term supplementary SCF treatment enhances elimination of CML ST-HSCs and MPPs but not primitive LT-HSCs in nilotinib-treated mice.

**Figure 8:**
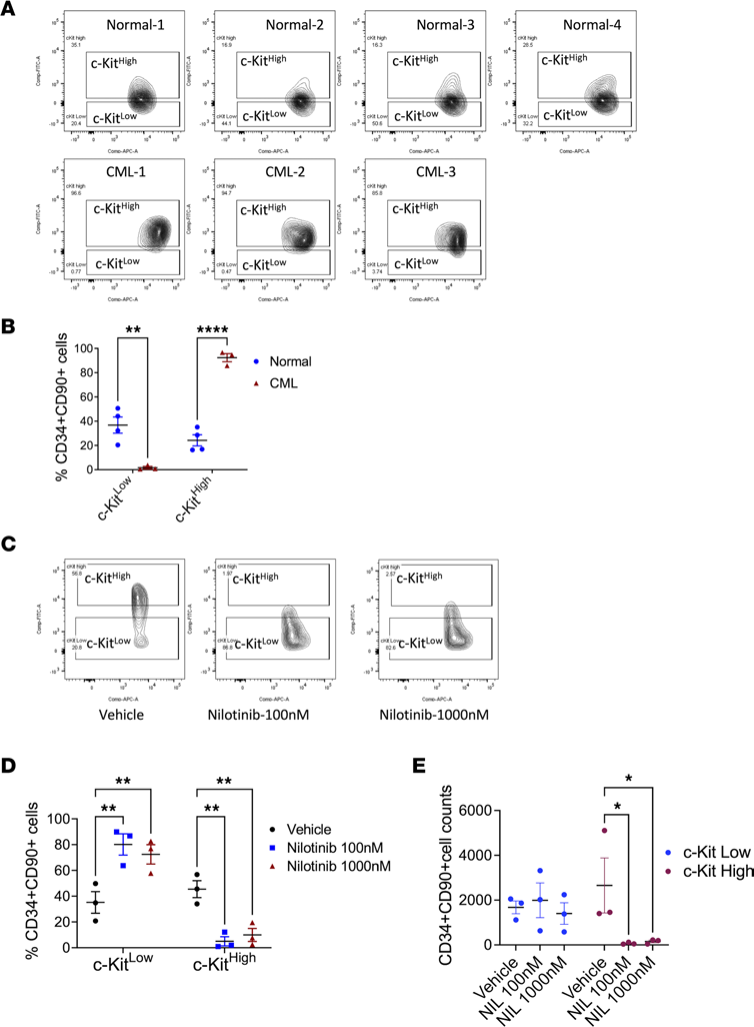
Effect of TKI treatment on human CML c-KIT^lo^ LT-HSCs. CD34^+^ cells from BM from patients with CML (*n* = 3) and healthy individuals (*n* = 4) were analyzed by flow cytometry, and LT-HSCs (CD34^+^CD38^–^CD90^+^) with high c-KIT expression (top 30%, c-KIT^hi^) and low c-KIT expression (bottom 30%, c-KIT^lo^) were identified, following the scheme used in Figure 1. Flow plots showing c-KIT expression in LT-HSCs are shown (**A**) with compiled data (**B**). CML CD34^+^ cells were cultured for 7 days in vitro without TKI (vehicle) or with nilotinib 100 nM and 1,000 nM and CD34^+^CD90^+^ cells were analyzed. Flow plots showing c-KIT expression are shown in **C** and compiled data for the proportion of c-KIT^lo^ and c-KIT^hi^ CD34^+^CD90^+^ cells in **D** and for number of c-KIT^lo^ and c-KIT^hi^ CD34^+^CD90^+^ cells in **E**. Data represented as mean ± SEM, *P < 0.05, **P < 0.01, ****P < 0.0001, based on 2-way ANOVA with Tukey’s test.

We further studied the effect of TKI treatment on human c-KIT^lo^ versus c-KIT^hi^ CML LT-HSCs. As with murine LT-HSCs, normal human LT-HSCs (CD34^+^CD38^–^CD90^+^) with the highest c-KIT expression (top 30%) were defined as c-KIT^hi^ LT-HSCs, and LT-HSCs with the lowest c-KIT expression (bottom 30%) were defined as c-KIT^lo^ LT-HSCs. Using the normal LT-HSC gates, increased proportions of human CML LT-HSCs were c-KIT^hi^ LT-HSCs, and reduced proportions were c-KIT^lo^ LT-HSCs, differing from our observations with murine stem cells (Figure 8, A and B). However, a significant reduction in proportions of c-KIT^hi^ CML LT-HSCs and increase in proportions of c-KIT^lo^ CML LT-HSCs were seen following culture of CML CD34^+^ cells with nilotinib for 7 days (Figure 8, C and D). CML c-KIT^hi^ LT-HSC numbers were significantly reduced whereas c-KIT^lo^ LT-HSC numbers were maintained after nilotinib treatment (Figure 8E). These results suggest that c-KIT^hi^ CML LT-HSCs were selectively depleted while c-KIT^lo^ CML LT-HSCs were preserved after TKI treatment.

## Discussion

TKI treatment does not eliminate primitive, quiescent CML LT-HSCs (2), which persist as a source of leukemia recurrence when treatment is discontinued. A subset of patients with CML can successfully maintain remission after stopping TKI treatment (40). However, long-term persistence of *BCR-ABL1*^+^ cells is observed, and early and late recurrences may be seen, indicating the importance of better understanding the heterogeneity of the regenerative and leukemogenic potential of CML LT-HSCs (41, 42). Here we identify variable expression of *c-Kit* as an important determinant of LT-HSC potential and TKI resistance in CML.

Previous studies showed that primitive, quiescent, self-renewing LT-HSCs are enriched within the c-KIT^lo^ subset in normal adult BM (21). Here we show that long-term repopulating and leukemia-initiating capacity is restricted to CML c-KIT^lo^ LT-HSCs, which are more quiescent, whereas CML c-KIT^hi^ LT-HSCs show increased cycling and lack repopulating potential. Therefore, CML c-KIT^lo^ LT-HSCs lie at the apex of the leukemia cell hierarchy. BCR-ABL was expressed and BCR-ABL signaling was active in CML c-KIT^lo^ LT-HSCs. CML LT-HSCs showed altered response to SCF stimulation with increased generation of progenitors and mature cells compared with LT-HSCs. SCF exposure was associated with reduced retention of LT-HSCs and increased generation of ST-HSCs and MPPs from CML c-KIT^lo^ LT-HSCs compared with normal c-KIT^lo^ LT-HSCs. Loss of SCF from perivascular and endothelial niches decreased normal HSC frequency and reconstitution ability (16). In contrast, deletion of SCF in CML mice reduced leukemia burden but increased retention of CML c-KIT^lo^ LT-HSCs. These observations suggest that SCF signaling promotes maturation of CML c-KIT^lo^ LT-HSCs toward c-KIT^hi^ LT-HSCs and expansion of their progeny. The effects of SCF deletion differed from those of deleting the chemokine CXCL12 from BM mesenchymal stem cells niches, a deletion that increases LSC cycling and self-renewal and accelerates leukemia development (39).

Heterogeneity in cell surface c-KIT expression on CML LT-HSCs was not related to differences in transcript levels. Previous studies also showed similar c-*Kit* mRNA expression in normal c-KIT^lo^ and c-KIT^hi^ HSCs (20). Cell surface c-KIT expression is regulated by activation and internalization of the protein after SCF binding, recruitment of signaling proteins, ubiquitination, and degradation (43). New c-KIT protein production is necessary for receptor reappearance on the cell surface. Reduced intracellular c-KIT expression in CML LT-HSCs may therefore reflect increased c-KIT protein degradation. Shin et al. showed a role for *c-Cbl*, an E3 ubiquitin ligase, in regulating c-KIT levels in LT-HSCs (21). Since c-CBL is prominently tyrosine-phosphorylated in BCR-ABL–expressing cells, reduced intracellular c-KIT levels could reflect altered c-CBL activity (44).

We report observations that CML c-KIT^hi^ LT-HSCs were depleted and CML c-KIT^lo^ LT-HSCs were maintained and enriched after TKI treatment. Importantly, these observations were validated with human CML LT-HSCs. These results indicate that CML LT-HSCs with low or absent c-KIT expression represent a TKI-resistant subpopulation and are consistent with the observation that primitive CD34^+^ cells that persist in CML patients on TKI treatment are characterized by lack of c-KIT expression (42). SCF stimulation failed to enhance LT-HSC cycling, and supplementary SCF treatment reduced CML ST-HSCs, MPPs, committed progenitors, and cell numbers in TKI-treated mice but failed to deplete CML LT-HSCs. Several BCR-ABL–targeting TKIs, including imatinib, nilotinib, dasatinib, and ponatinib, have c-KIT–inhibitory activity in addition to ABL kinase–inhibitory activities (45). However, Corbin at al. reported that c-KIT inhibition by TKI contributes to effects on CML progenitors but not stem cells (46).

CML c-KIT^lo^ LT-HSCs expressed gene signatures for inflammatory signaling and quiescence, which are also characteristic of LT-HSC subpopulations that persist in patients with CML following TKI treatment (38). CML c-KIT^lo^ LT-HSCs were also enriched for signatures for Wnt and Hedgehog signaling, important developmental regulators for CML LSC maintenance and self-renewal (5, 34, 36, 37). Finally, CML c-KIT^lo^ LT-HSCs showed altered metabolic gene signatures, with increased glycolytic but reduced oxidative and fatty acid metabolism gene signatures. CML primitive progenitors are reported to have enhanced OXPHOS and to be sensitive to mitochondrial inhibitors (47). The present studies indicate differences in metabolic gene signatures among primitive CML LT-HSCs with high and low c-KIT expression and suggest that heterogeneous LT-HSC subpopulations may have different metabolic dependencies.

In conclusion, our results provide improved resolution of the heterogeneity of CML LT-HSC populations based on different levels of c-KIT expression. Leukemia-initiating cells with in vivo regenerating capacity are enriched within the CML c-KIT^lo^ LT-HSC population and depleted within the c-KIT^hi^ LT-HSC population. c-KIT^lo^ LT-HSCs are resistant to TKIs and are enriched after TKI treatment. Human CML LT-HSCs with low or absent c-KIT expression are enriched after exposure to TKIs, consistent with the observation that CML stem cells persisting in TKI-treated patients with CML lack c-KIT expression, supporting the clinical relevance of our work. Thus, our studies identify and characterize a quiescent, treatment-resistant, primitive leukemia-initiating subpopulation in CML that is an important target for further studies aimed at eliminating persistent disease.

## Methods

### Animal studies

UBC-cre (B6.Cg-*Ndor1^Tg(UBC-cre/ERT2)1Ejb^*/1J), Scf^Δ/Δ^ (*Kitl^tm2.1Sjm^*/J), Scf^gfp/+^ (*Kitl^tm1.1Sjm^*/J), and C57BL/6 mice were from The Jackson Laboratory, and C57BL/6.SJL mice from Charles River Laboratories. Scl-tTA-BCR-ABL mice were maintained on doxycycline chow (Envigo). For transplantation, mice were lethally irradiated at 4 Gy times 2 doses, 4 hours apart, and total BM or FACS-sorted LT-HSCs were transplanted along with 2 × 10^5^ support BM cells via intravenous injection. Mice received sulfatrim food (TestDiet) posttransplantation. Experiments were performed using mice of both sexes at 6–12 weeks old. Mice were randomly divided into experimental groups. Mice were subjected to 12-hour light/12-hour dark cycles and controlled ambient room temperature and humidity. All mice were maintained in Association for Assessment and Accreditation of Laboratory Animal Care International–accredited, specific pathogen–free animal care facilities, and procedures were conducted in accordance with federal guidelines using protocols approved by the Institutional Animal Care and Use Committee at the University of Alabama at Birmingham (UAB). Animal models utilized in this study are summarized in the Supplemental Methods.

### Human samples

BM and PB were obtained from patients with CML seen at the UAB. Normal PB stem cells were obtained from transplant donors also recruited at the UAB. Mononuclear cells were isolated by Ficoll-Hypaque (Sigma Diagnostics) centrifugation. CD34^+^ cells were isolated using immunomagnetic beads (Miltenyi Biotec). Sample acquisition was approved by the UAB Institutional Review Board in accordance with assurances filed with the Department of Health and Human Services and requirements of the Declaration of Helsinki. Informed consent was obtained from patients and healthy donors.

### Drug administration

Tamoxifen (75 mg/kg, Cayman Chemical) in corn oil was administered intraperitoneally for 5 days to Ubc-Scf^Δ/Δ^-cre mice. Nilotinib (50 mg/kg/day in 0.5% methylcellulose + 0.5% Tween-80, Novartis) was administered by oral gavage. SCF (100 μg/kg, PeproTech) was administered intraperitoneally for 2 weeks. For in vitro analysis, cells were cultured with 5 μM nilotinib, 10 ng/mL SCF, or combination, for 7 days.

### Flow cytometry

BM cells were isolated by crushing in PBS with 2% heat-inactivated fetal bovine serum (Thermo Fisher Scientific). Spleen cells were obtained by crushing and filtering through a 70 μm filter mesh (Thermo Fisher Scientific). PB, BM, and spleen cells were labeled with relevant antibodies as summarized in the Supplemental Methods, including anti-CD45.1, anti-CD45.2, anti–Gr-1, anti–Mac-1, anti-B220, anti-CD19, and anti-CD3; lineage cocktail (anti-ter119, anti-CD3e, anti-Nk1.1, anti-CD4, anti-CD19, anti–Gr-1, anti–Mac-1, anti-B220, anti-CD8a, and anti-CD8b); and anti–c-KIT (2B8), anti–Sca-1, anti-Flt3, anti-CD150, anti-CD48, anti-CD127, anti-CD16/32, anti-CD34, anti-CD105, anti-CD41, anti-CD71, and anti-Ter119. For transplanted mice, donor cells were selected based on CD45.2 or CD45.1/2 expression as appropriate. Mature populations were identified as follows: myeloid cells (Gr-1^+^Mac-1^+^), B cells (B220^+^CD19^+^), and T cells (CD3^+^). Hematopoietic stem/progenitor populations included GMPs (Lin^–^Sca-1^–^c-KIT^+^CD34^+^FcgR^+^), MEPs (Lin^–^Sca-1^–^c-KIT^+^CD34^–^FcgR^–^), MPPs (Lin^–^Sca-1^+^c-KIT^+^Flt3^–^CD48^+^), ST-HSCs (Lin Sca-1^+^c-KIT^+^Flt3^–^CD150^–^CD48^–^), and LT-HSCs (Lin^–^Sca-1^+^c-KIT^+^Flt3^–^CD150^+^CD48^–^). To assess BM stromal populations, long bones were crushed, and fragments were digested with collagenase II (50 U/mL, MilliporeSigma), dispase II (200 U/mL, MilliporeSigma), and DNase I (100 U/mL, MilliporeSigma); washed; and mixed with the BM supernatant. Cells were labeled with anti-CD45, anti-Ter119, anti-CD31, anti-CD51, anti–Sca-1, and anti-CD140a antibodies and DAPI/Aqua blue (Thermo Fisher Scientific) or Fixable Dye eFluor 450. Cells were analyzed on LSR Fortessa, FACS LSR II, or FACSAria II (BD Biosciences) and data processed using FlowJo LLC.

For cell cycle analysis, BM cells were stained for stem cell markers, fixed using BD Cytofix/Cytoperm (BD Biosciences), and then stained with Ki-67 and DAPI and analyzed using flow cytometry. For intracellular flow cytometry for p-STAT5, lineage-negative cells were enriched from BM using lineage microbeads and the autoMACS Pro Separator (both Miltenyi Biotec), labeled with antibodies for stem cell markers, fixed with 4% paraformaldehyde, permeabilized with Cytofix/Cytoperm, and stained with p-STAT5–PE (1:50 dilution, Cell Signaling Technology).

### In vitro culture

#### Murine cells.

Immature cells were enriched from BM using lineage microbeads and the autoMACS Pro Separator and stained with antibodies against lineage markers; anti-Flt3, anti–c-KIT, anti–Sca-1, anti-CD150, and anti-CD48 (Thermo Fisher Scientific); and DAPI. Samples were sorted using a FACSAria II. Cells were cultured in U-bottom, 96-well plates (100 cells/well) in StemSpan SFEM II medium (StemCell Technologies) with penicillin/streptomycin (Invitrogen, Thermo Fisher Scientific), antibiotic/antimycotic (HyClone), and erythropoietin, thrombopoietin (TPO), IL-3, IL-6, FLT3, GMSCF (all 10 ng/mL; PeproTech), and SCF at 37°C and 5% CO_2_. Cell populations were enumerated by adding Countbright beads (Thermo Fisher Scientific); Lin^–^ (anti-ter119, anti-IgG, anti-CD3e, anti-Nk1.1, anti-CD4, anti-CD19, anti–Gr-1, anti-B220, anti-CD8a, and anti-CD8b); anti-CD11b, anti–Sca-1, anti-CD117, anti-Flt3, anti-CD150, anti-CD48, and DAPI or Fixable Dye eFluor 450 to wells and analyzed on an LSR Fortessa.

#### Human cells.

Human CD34^+^ cells were cultured in StemSpan SFEM II media supplemented with human IL-3 (20 ng/mL), G-CSF (20 ng/mL), FLT3 ligand (50 ng/mL), TPO (25 ng/mL), SCF (25 ng/mL) (PeproTech), SR1 (500 nM), UM729 (1 μM, IRIC), penicillin, and glutamine for 7 days with or without TKI (nilotinib 100 nM, 1,000 nM).

### SCF ELISA

BM was extracted from femurs in 100 μL of PBS; cells were pelleted at 1,000*g* for 10 minutes at 4°C; and supernatant containing BM plasma was obtained. SCF concentrations were analyzed using a mouse Quantikine SCF ELISA Kit (R&D Systems, Bio-Techne).

### Genomic PCR and qRT-PCR

Genomic PCR was performed as described in Ding et al. (16). For qRT-PCR, RNA was extracted (Qiagen) and converted to cDNA (Invitrogen, Thermo Fisher Scientific). *c-KIT* (Mm00445212_m1) and SCF (Mm00442972_m1) expression were measured using TaqMan probes (Invitrogen, Thermo Fisher Scientific) using a QuantStudio 6 (Thermo Fisher Scientific). Expression levels were normalized to GAPDH (Mouse GAPDH VIC-MGB, Applied Biosystems, Thermo Fisher Scientific). For *Bcr-Abl1* mRNA measurements, RNA was extracted from c-KIT^lo^ and c-KIT^hi^ cells LT-HSCs using the RNeasy Plus Micro Kit (Qiagen), cDNA was synthesized using the Superscript III first strand kit (Invitrogen, Thermo Fisher Scientific), and qRT-PCR was performed using TaqMan universal PCR master mix kit and the QuantStudio 6 Flex (Applied Biosystems, Thermo Fisher Scientific). Primer and probe sequences for B3A2 were as previously described (48).

### RNA-Seq analysis

RNA was extracted from normal and leukemic BM c-KIT^lo^ and c-KIT^hi^ LT-HSCs using RNeasy Plus Micro Kit (Qiagen), with 4 biological replicates per group. Sequencing libraries were prepared with the SMARTer Ultra Low Input RNA Kit for Sequencing (v4, TaKaRa) and Nextera XT DNA Library Preparation Kit (96 samples, Illumina). Sequencing was performed using the HiSeq 2500 platform with the HiSeq SBS Kit V4 (Illumina). STAR (version 2.5.3a) was used to align RNA-Seq FASTQ reads to the mouse reference genome (Gencode Release M11), and reads mapping to each gene were enumerated using HTSeq-count50. Normalization and differential expression analysis was done using DESeq2 (Bioconductor) (49). Volcano plots were created using the R statistical program. Enrichment of gene signatures was analyzed using GSEA, Broad Institute (50). Protein-protein association network analysis was performed using STRING (STRINGv11) (51). Heatmaps were created using Heatmapper (52).

### Data sharing

The RNA-Seq data have been deposited in the NCBI’s Gene Expression Omnibus (accession number GSE180496) (https://www.ncbi.nlm.nih.gov/geo/query/acc.cgi?acc=GSE180496). For original data, please contact the corresponding author at r.bhatia@uabmc.edu.

### Statistics

Unless otherwise specified, results obtained from independent experiments are reported as means ± SEM of multiple replicates, and statistical analyses were performed using unpaired, nonparametric, 2-tailed *t* test or 2-way ANOVA, adjusting for multiple comparisons with Tukey’s test or Holm-Šídák test as indicated (GraphPad Prism version 6.0). Data were examined for normality by evaluating skewness and for equivalence of variance by comparing variance between groups. *P* values less than 0.05 were considered statistically significant.

### Study approval

All mouse experiments were approved by the Institutional Animal Care and Use Committee at the UAB. Studies using human samples were approved by the UAB Institutional Review Board in accordance with assurances filed with the Department of Health and Human Services. Written informed consent was obtained from patients and healthy donors.

## Author contributions

MS designed, planned, and conducted experiments; analyzed data; and wrote the manuscript. HK and SQ designed, planned, and conducted experiments; HL, MH, and AA conducted experiments; JH and DKC analyzed the data; and AP and RSW reviewed and edited the manuscript. RB designed the experiments, analyzed data, and wrote the manuscript.

## Supplementary Material

Supplemental data

## Version 1 11/22/2022

In-Press Preview

## Version 2 01/10/2023

Electronic publication
