# Supplementary material for: Low c-Kit expression identifies primitive, therapy-resistant CML stem cells
Source: JCI Insight. 2023 Jan 10;8(1):e157421. doi: 10.1172/jci.insight.157421 (PMC9870079; doi:10.1172/jci.insight.157421)
Supplement: Supplemental data [file jciinsight-8-157421-s067.pdf]

Supplementary Information

Supplementary Methods Table

REAGENT or RESOURCE	SOURCE	IDENTIFIER
**Antibodies**		
Anti-mouse CD3	Thermo Fisher Scientific	Cat#13-0031-85; RRID:AB_466320
Anti-mouse CD4	Thermo Fisher Scientific	Cat#13-0041-85; RRID:AB_466326
Anti-mouse CD8	Thermo Fisher Scientific	Cat#13-0083-85; RRID:AB_657763
Anti-mouse B220	Thermo Fisher Scientific	Cat#13-0452-85; RRID:AB_466450
Anti-mouse CD19	Thermo Fisher Scientific	Cat#13-0193-85; RRID:AB_657658
Anti-mouse IgM	Thermo Fisher Scientific	Cat#13-5790-85; RRID:AB_466676
Anti-mouse Gr-1	Thermo Fisher Scientific	Cat#13-5931-85; RRID:AB_466801
Anti-mouse CD11b	Thermo Fisher Scientific	Cat#13-0112-85; RRID:AB_466360
Anti-mouse NK1.1	Thermo Fisher Scientific	Cat#13-5941-85; RRID:AB_466805
Anti-mouse Ter119	Thermo Fisher Scientific	Cat#13-5921-85; RRID:AB_466798
Anti-mouse CD45.1	Thermo Fisher Scientific	Cat#25-0453-82; RRID:AB_469629
Anti-mouse CD45.2	Thermo Fisher Scientific	Cat#11-0454-85; RRID:AB_465062
Anti-mouse CD3	Thermo Fisher Scientific	Cat#100218; RRID:AB_1595492
Anti-mouse CD19	Thermo Fisher Scientific	Cat#17-0193-82; RRID:AB_1659676
Anti-mouse Ter119	Thermo Fisher Scientific	Cat#116220; RRID:AB_528963
Anti-mouse B220	Thermo Fisher Scientific	Cat#56-0452-82; RRID:AB_891458
Anti-mouse CD11b	Thermo Fisher Scientific	Cat#47-0112-82; RRID:AB_1603193
Anti-mouse Gr-1	Thermo Fisher Scientific	Cat#12-5931-83; RRID:AB_466046
Streptavidin	BioLegend	Cat#405229
Anti-mouse CD45	Thermo Fisher Scientific	Cat#56-0451-82; RRID:AB_891454
Anti-mouse CD16/32	Thermo Fisher Scientific	Cat#56-0161-82; RRID:AB_493994
Anti-mouse CD117	Biolegend	ca#105826; RRID:AB_1626278
Anti-mouse Sca-1	Biolegend	Cat#12-5981-82; RRID:AB_493596
Anti-mouse CD48	Thermo Fisher Scientific	Cat#17-0481-82; RRID:AB_469408
Anti-mouse CD150	BioLegend	Cat#115922; RRID:AB_2303663
Anti-mouse CD150	Thermo Fisher Scientific	Cat#12-1502-82; RRIDAB_1548765
Anti-mouse CD34	Thermo Fisher Scientific	Cat#50-0341-82; RRID:AB_10596826
Anti-mouse Flt3	Thermo Fisher Scientific	Cat# 15-1351-82, RRID:AB_494219
Anti-mouse CD127	BD Biosciences	Cat# 562419, RRID:AB_11153131
Anti-mouse CD105	Biolegend	Cat# 120411, RRID:AB_1877185
Anti-mouse CD41	BD Biosciences	Cat# 740136, RRID:AB_2739892
Anti-mouse CD71	BD Biosciences	Cat# 553266, RRID:AB_394743
Anti-mouse Ter119	Thermo Fisher Scientific	Cat# 15-5921-82, RRID:AB_468810
Anti-mouse CD45.1	BioLegend	Cat# 110727, RRID:AB_893348
Anti-mouse CD45.2	Thermo Fisher Scientific	Cat# 17-0454-82, RRID:AB_469400
Anti-mouse CD45.1	BioLegend	Cat# 110723, RRID:AB_493732
Anti-Ki67	Thermo Fisher Scientific	Cat#11-5698-80; RRID:AB_11151689
Phospho-Stat5 (Tyr694)	Cell Signaling Technology (CST)	Cat# 5387
Anti-human CD45	Thermo Fisher Scientific	Cat # 47-0459-42; RRID:AB_1944368
Anti-human CD34	Thermo Fisher Scientific	Cat# 17-0349-42; RRID:AB_2016672
Anti-human CD38	Thermo Fisher Scientific	Cat # 12-0388-42; RRID:AB_1518748
Anti-human CD90	Thermo Fisher Scientific	Cat # 45-0909-42; RID:AB_10718245

Anti-human CD45RA	Thermo Fisher Scientific	Cat # 25-0458-42; RRID:AB_1548774
Anti-human CD117 (c-Kit)	Thermo Fisher Scientific	Cat # 11-1178-42; RRID:AB_2572472
**Chemicals, Peptides, and Recombinant Proteins**		
Collagenase II	Sigma-Aldrich	Cat# C1764
Dispase II	Sigma-Aldrich	Cat#4942078001
DNase I	Sigma-Aldrich	Cat#D4263
BSA	Sigma-Aldrich	Cat#A9418
DAPI	Thermo Fisher Scientific	Cat#D1306; RRID:AB_2629482
Fixable Viability Dye eFluor™ 450	Thermo Fisher Scientific	Cat#65-0863-14
**Critical Commercial Assays**		
Quantikine SCF Elisa Kit	R&D	cat# MCK00
RNeasy Micro Kit	QIAGEN	Cat#74004
Direct Lineage Cell Depletion Kit	Miltenyi Biotech	Cat#130-110-470
Nextera XT DNA Library Preparation Kit	Illumina	Cat#FC-131-1096
SMART-Seq v4 Ultra Low Input RNA Kit	Takara Bio	Cat#634891
**Deposited Data**		
RNA Seq data	UAB Bioinformatics Core	GSE180496
**Experimental Models: Organisms/Strains**		
Mouse: SCL-tTA-BCR-ABL	Daniel Tenen, BIDMC, Boston	N/A
Mouse: B6.SJL-PtprcaPepcb/BoyCrCrl	Charles River Laboratory	Cat#564
Mouse: C57BL/6NCr	Charles River Laboratory	Cat#556
Mouse: ScfΔ/Δ (Kitltm2.1Sjm/J)	Jackson Laboratory	Stock No: 017861
Mouse: Scfgfp/+ (Kitltm1.1Sjm/J),	Jackson Laboratory	Stock No: 017860
**Oligonucleotides**		
mKitl qPCR probe	Thermo Fisher Scientific	Cat#Mm00442972_m1
mKit qPCR probe	Thermo Fisher Scientific	Cat#Mm00445212_m1
mGapdh qPCR probe	Thermo Fisher Scientific	Cat#Mm99999915_g1
**Software and Algorithms**		
FlowJo software (version 8.5.2)	FlowJo	RRID:SCR_008520
BD FACSDiva	BD Biosciences	RRID:SCR_001456
GraphPad Prism 7	GraphPad	RRID:SCR_002798

Supplementary Table 1. GSEA analysis using the C2 gene set

CML c-Kit^{Low} vs c-Kit^{High} (positive correlation)	**NES**	**FDR q-val**
**TNFA/NF-KB pathway**		
PID_TNF_PATHWAY	2.116324	0
BIOCARTA_NFKB_PATHWAY	1.907848	0.006071145
SCHEIDEREIT_IKK_INTERACTING_PROTEINS	1.849322	0.007142454
BIOCARTA_TNFR2_PATHWAY	1.747719	0.015076728
REACTOME_NFKB_AND_MAP_KINASES_ACTIVATION_MEDIATED_BY_TLR4_SIGNALING_REPERTO	1.741105	0.0157799
REACTOME_TRAF6_MEDIATED_INDUCTION_OF_NFKB_AND_MAP_KINASES_UPON_TLR7_8_OR_	1.720655	0.018181676
ZHANG_RESPONSE_TO_IKK_INHIBITOR_AND_TNF_UP	1.694828	0.022621538
**IL1/IL2/IL6 signaling**		
REACTOME_SIGNALING_BY_ILS	1.929466	0.004898063
BIOCARTA_IL22BP_PATHWAY	1.886121	0.006695281
BIOCARTA_IL1R_PATHWAY	1.876359	0.006694058
PID_IL2_1PATHWAY	1.872696	0.006694001
REACTOME_IL_3_5_AND_GM_CSF_SIGNALING	1.863667	0.007030382
BYSTROEM_CORRELATED_WITH_IL5_DN	1.86354	0.006870613
PID_IL6_7_PATHWAY	1.826958	0.008023258
PID_IL1_PATHWAY	1.792247	0.010340108
SIG_IL4RECEPTOR_IN_B_LYPHOCYTES	1.773158	0.011945327
REACTOME_IL1_SIGNALING	1.72922	0.016938413
ST_INTERLEUKIN_4_PATHWAY	1.717131	0.018580658
BIOCARTA_IL2_PATHWAY	1.709963	0.019836994
BIOCARTA_IL6_PATHWAY	1.708532	0.020122023
WIERENGA_STAT5A_TARGETS_DN	1.604818	0.04359974
**WNT signaling**		
BIOCARTA_WNT_PATHWAY	1.84486	0.006766611
PID_WNT_CANONICAL_PATHWAY	1.844276	0.006712065
KEGG_WNT_SIGNALING_PATHWAY	1.655055	0.031312972
ST_WNT_BETA_CATENIN_PATHWAY	1.601411	0.044222824

CML c-Kit^{Low} vs c-Kit^{High} (negative correlation)	**NES**	**FDR q-val**
**Oxidative Phosphorylation**		
MOOTHA_VOXPHOS	-2.8651743	0
KEGG_OXIDATIVE_PHOSPHORYLATION	-2.8302815	0
REACTOME_TCA_CYCLE_AND_RESPIRATORY_ELECTRON_TRANSPORT	-2.3308887	0
MOOTHA_MITOCHONDRIA	-1.8251351	0.013205438
**Cell proliferation**		
REACTOME_REGULATION_OF_MITOTIC_CELL_CYCLE	-2.2418208	2.14E-05
REACTOME_CYCLIN_E_ASSOCIATED_EVENTS_DURING_G1_S_TRANSITION_	-2.2367766	4.35E-05
GRAHAM_CML_DIVIDING_VS_NORMAL_QUIESCENT_UP	-1.9096578	0.006530864
ZHOU_CELL_CYCLE_GENES_IN_IR_RESPONSE_24HR	-1.8824551	0.008336465
REACTOME_S_PHASE	-1.8561777	0.010300171
REACTOME_M_G1_TRANSITION	-1.7986846	0.016241971
REACTOME_G1_S_TRANSITION	-1.7903509	0.017485164
GRAHAM_NORMAL_QUIESCENT_VS_NORMAL_DIVIDING_DN	-1.7653209	0.020875871
REACTOME_DNA_REPLICATION	-1.7505176	0.023024708
REACTOME_CELL_CYCLE_CHECKPOINTS	-1.7328736	0.025807915
REACTOME_MITOTIC_M_M_G1_PHASES	-1.7038441	0.031561214
REACTOME_CELL_CYCLE	-1.7027274	0.031628624
TANG_SENESCENCE_TP53_TARGETS_DN	-1.6922445	0.034107182
ISHIDA_E2F_TARGETS	-1.6921725	0.033888545
REACTOME_CELL_CYCLE_MITOTIC	-1.6510344	0.043265298
KONG_E2F3_TARGETS	-1.6403509	0.04547825
WHITFIELD_CELL_CYCLE_LITERATURE	-1.5879381	0.060423955

CML c-Kit^{Low} vs Normal c-Kit^{Low} (positive correlation)	**NES**	**FDR q-val**
**Cell cycle**		
EGUCHI_CELL_CYCLE_RB1_TARGETS	2.041842	0.00312
PID_E2F_PATHWAY	2.00988	0.00378
KONG_E2F3_TARGETS	1.999559	0.004074
CHANG_CYCLING_GENES	1.953056	0.006972
ROSTY_CERVICAL_CANCER_PROLIFERATION_CLUSTER	1.870724	0.016825
REICHERT_MITOSIS_LIN9_TARGETS	1.832064	0.022796
WHITFIELD_CELL_CYCLE_LITERATURE	1.809162	0.02946
TANG_SENESCENCE_TP53_TARGETS_DN	1.716452	0.055583
ISHIDA_E2F_TARGETS	1.710974	0.056271
FISCHER_G2_M_CELL_CYCLE	1.671032	0.068208
SCIAN_CELL_CYCLE_TARGETS_OF_TP53_AND_TP73_DN	1.642611	0.077356
REN_BOUND_BY_E2F	1.635987	0.076771
**Inflammation**		
BIOCARTA_IL1R_PATHWAY	1.877218	0.016093
MANTOVANI_NFKB_TARGETS_UP	1.850237	0.020109
PHONG_TNF_TARGETS_UP	1.800883	0.032767
GALINDO_IMMUNE_RESPONSE_TO_ENTEROTOXIN	1.776825	0.038194
BIOCARTA_TNFR1_PATHWAY	1.765925	0.03964
SCHOEN_NFKB_SIGNALING	1.719057	0.055504
CROONQUIST_IL6_DEPRIVATION_DN	1.717999	0.055546
DASU_IL6_SIGNALING_SCAR_UP	1.706593	0.057743
BIOCARTA_NFKB_PATHWAY	1.687181	0.06174
MAHAJAN_RESPONSE_TO_IL1A_UP	1.664432	0.070661
TIAN_TNF_SIGNALING_NOT_VIA_NFKB	1.654689	0.077293
ZHOU_INFLAMMATORY_RESPONSE_FIMA_UP	1.636054	0.077534

CML c-Kit^{Low} vs Normal c-Kit^{Low} (negative correlation)	NES	FDR q-val
**Oxidative phosphorylation**		
MOOTHA_VOXPHOS	-2.6092	0
WONG_MITOCHONDRIA_GENE_MODULE	-2.56294	0
REACTOME_TCA_CYCLE_AND_RESPIRATORY_ELECTRON_TRANSPORT	-2.47558	0
KEGG_OXIDATIVE_PHOSPHORYLATION	-2.32705	7.89E-05
MOOTHA_MITOCHONDRIA	-1.94712	0.006495
MOOTHA_PGC	-1.55193	0.134756
**DNA Damage**		
REACTOME_P53_INDEPENDENT_G1_S_DNA_DAMAGE_CHECKPOINT	-2.22717	2.27E-04
REACTOME_P53_DEPENDENT_G1_DNA_DAMAGE_RESPONSE	-2.19736	2.28E-04
**interferon**		
ZHANG_INTERFERON_RESPONSE	-2.18809	2.82E-04
REACTOME_INTERFERON_ALPHA_BETA_SIGNALING	-1.99925	0.003644
RADAEVA_RESPONSE_TO_IFNA1_UP	-1.8887	0.012022
BROWNE_INTERFERON_RESPONSIVE_GENES	-1.88273	0.012581
DER_IFN_ALPHA_RESPONSE_UP	-1.79038	0.029329
MOSERLE_IFNA_RESPONSE	-1.76144	0.036749
REACTOME_INTERFERON_SIGNALING	-1.61588	0.093382
BOSCO_INTERFERON_INDUCED_ANTIVIRAL_MODULE	-1.57393	0.1226
DER_IFN_GAMMA_RESPONSE_UP	-1.48814	0.170784
SANA_RESPONSE_TO_IFNG_UP	-1.46686	0.186366

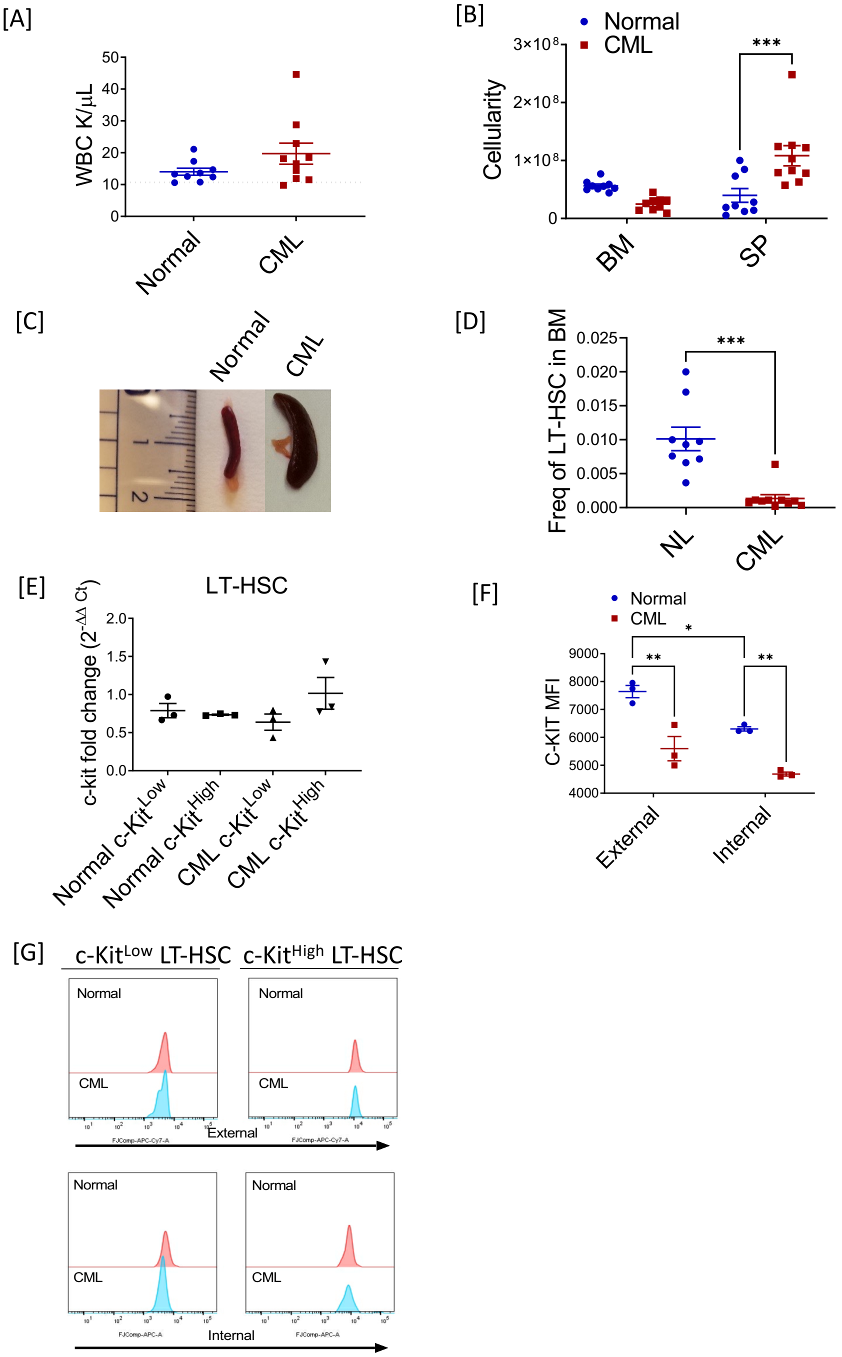

Supplementary Figure 1. C-Kit^{Low} LT-HSCs are increased in CML compared to normal bone marrow. (Related to Figure 1). Peripheral blood WBC [A], bone marrow cellularity [B] and spleen size [C] in normal control and CML-induced mice. Frequency of donor LT-HSCs of normal control and CML-induced mice [D]. Expression of c-Kit mRNA measured by Q-PCR (2-ΔΔcT values) in normal and CML c-Kit^{Low} and c-Kit^{High} LT-HSC. Data normalized to GAPDH (n=3) [E]. MFI of cell surface and internal c-Kit levels in normal and CML LT-HSCs [F]. Histograms of internal and external c-Kit expression from concatenated normal and CML LT-HSCs stratified as c-Kit^{Low} and c-Kit^{High} LT-HSCs (n=3 mice) [G]. Data represented as mean ± SEM, *p<0.05; **p<0.01, ***p<0.001.

[A]

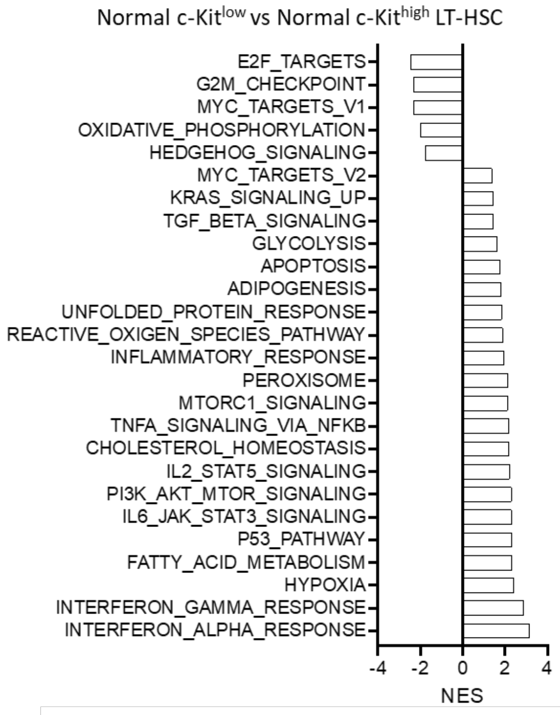

[B]

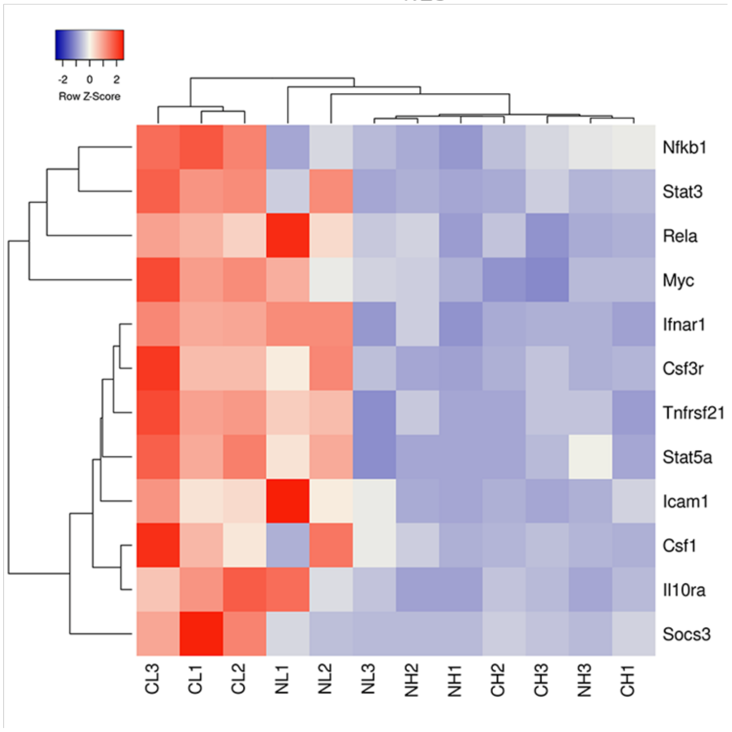

Supplementary Figure 2. CML c-Kit^{Low} LT-HSC exhibit gene signatures characteristic of primitive drug resistant leukemia stem cells (Related to Figure 2) Normalized enrichment scores (NES) of Hallmark gene sets significantly enriched (FDR<0.05) in Normal c-Kit^{Low} LT-HSCs compared to Normal c-Kit^{High} LT-HSCs [A]. Heatmap showing inflammatory gene expression in Normal and CML c-Kit^{Low} LT-HSCs and c-Kit^{High} LT-HSCs [B].

[A]

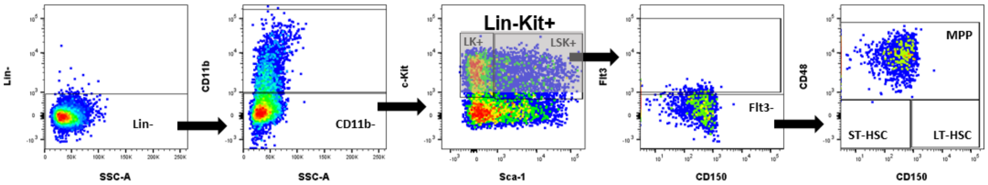

Supplementary Figure 3. Differential response of Normal and CML LT-HSC to SCF. (Related to Figure 4)
A. Gating strategy of cultured mouse stem/progenitor cells.

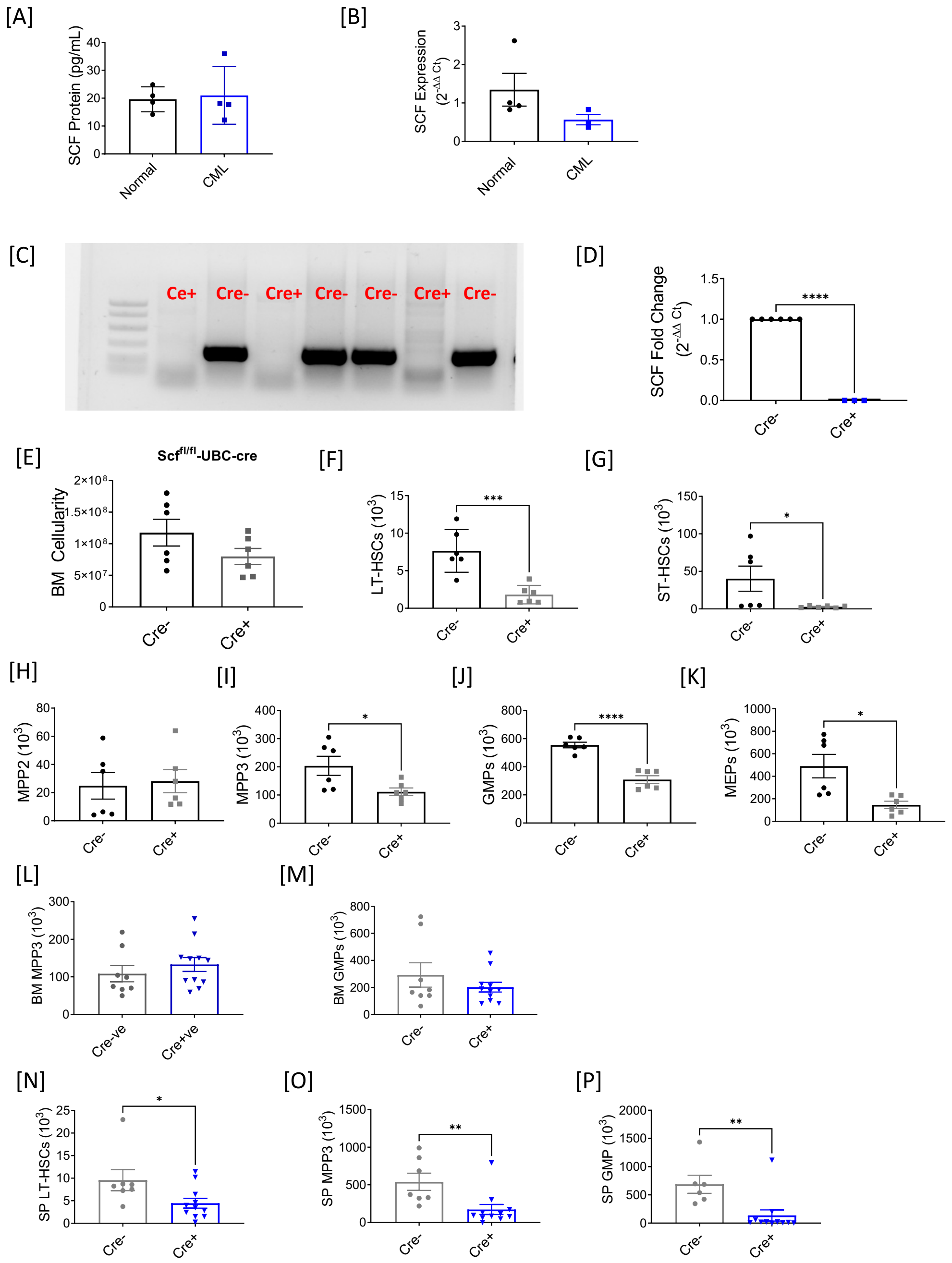

Supplementary Figure 4. **Deletion of SCF alters c-Kit^{Low} LT-HSC numbers in vivo.** (Related to Figure 6) ELISA for SCF protein in BM lysates [A], and q-RT-PCR for SCF mRNA expression normalized to GAPDH (2-ΔΔcT values) in whole murine bone marrow cells, from normal and SCL-tTA-BCR-ABL mice (n=4) [B]. Genomic PCR for Scf excision [C] and q-RT-PCR for Scf mRNA expression in BM cells [D] from cre- and cre+ Scf$^{fl/fl}$/Ubc-cre mice. Total number of bone marrow cells [E], LT-HSC [F], ST-HSCs [G], MPP2 [H], MPP3 [I], GMP [J], and MEP [K] cells in cre- (n=3-6) and cre+ (n=3-6) Scf$^{fl/fl}$/Ubc-cre mice. Total number of donor bone marrow MPP3 [L], and GMP [M], and donor spleen LT-HSCs [N], MPP3 [O], and GMP [P], 14 weeks post transplantation of CML bone marrow cells into lethally irradiated cre- (n=3-6) and cre+ (n=8-11) Scf$^{fl/fl}$/Ubc-cre mice. Data represented as mean ± SEM, *$p<0.05$; **$p<0.01$, ***$p<0.001$, ****$p<0.0001$

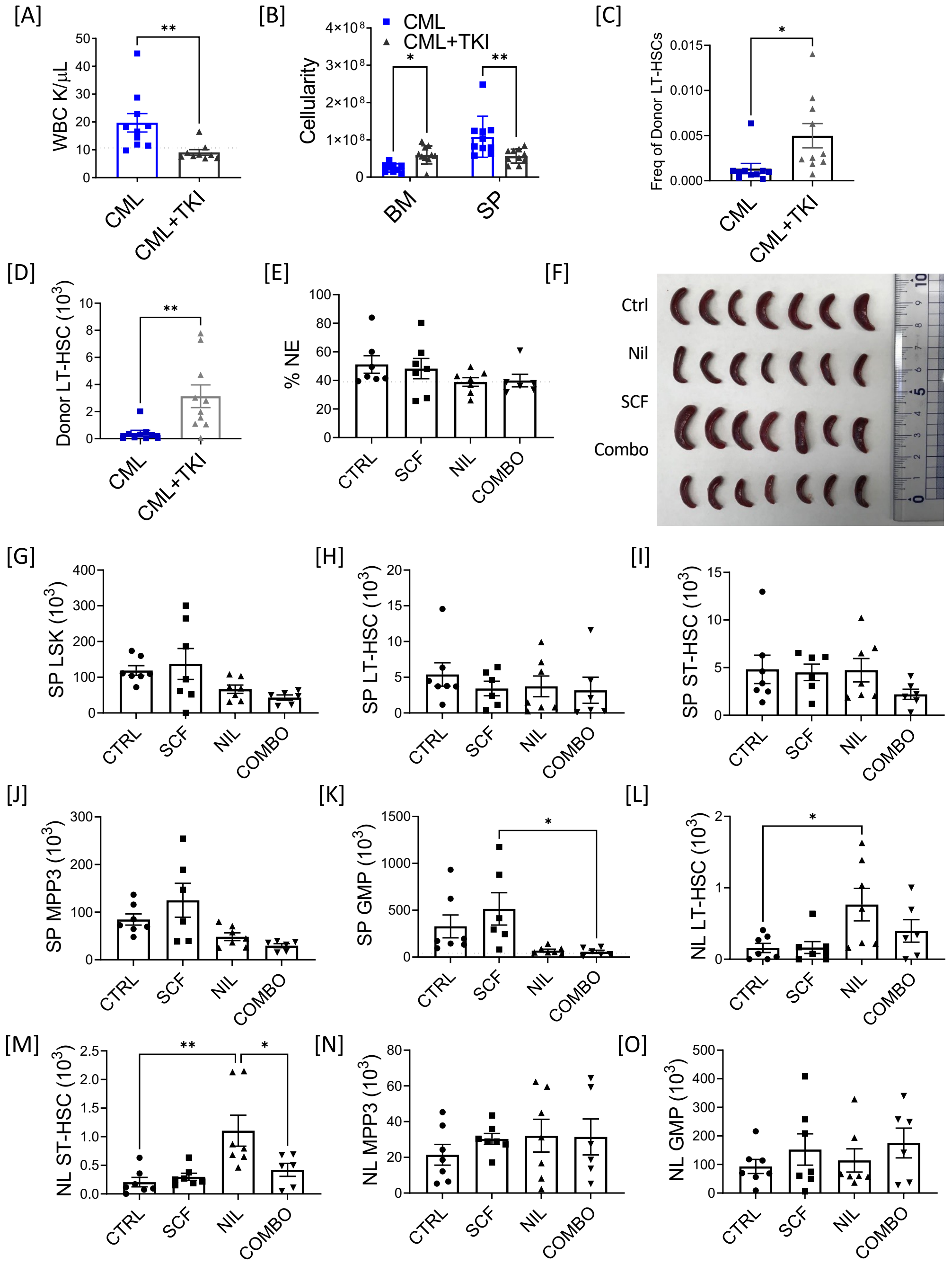

Supplementary Figure 5. **Effect of TKI-treatment on murine leukemic LT-HSC** (Related to Figure 7) Peripheral blood WBC [A] and bone marrow cellularity [B] in CML mice with and without nilotinib (TKI)-treatment. The dotted line represents the upper limit of normal range. Frequency of CML LT-HSC [C], and number of CML LT-HSC [D] in CML mice with and without nilotinib (TKI) treatment. CML-induced mice were treated with SCF, Nilotinib, or Combo for 14 days. Percentage of Neutrophils [E] in the blood post treatment. Spleen size [F], and total number donor CML spleen LSK [G], LT-HSC [H], ST-HSC, [I], MPP3, [J] and GMP[K]. Total number of normal donor bone marrow LT-HSC [L], ST-HSCs [M], MPP3 [N], GMP [O], of control (CRTL), SCF-treated (SCF), Nilotinib-treated (NIL) or SCF and Nilotinib-treated (COMBO) mice. n=6-7. Data represented as mean ± SEM, *p<0.05, **p<0.01.
